# Water-Exchange-Modified Kinetic Parameters from Dynamic Contrast-Enhanced MRI as Prognostic Biomarkers of Survival in Advanced Hepatocellular Carcinoma Treated with Antiangiogenic Monotherapy

**DOI:** 10.1371/journal.pone.0136725

**Published:** 2015-09-14

**Authors:** Sang Ho Lee, Koichi Hayano, Andrew X. Zhu, Dushyant V. Sahani, Hiroyuki Yoshida

**Affiliations:** 1 3D Imaging Research, Department of Radiology, Massachusetts General Hospital and Harvard Medical School, Boston, Massachusetts, United States of America; 2 Division of Abdominal Imaging and Intervention, Department of Radiology, Massachusetts General Hospital, Boston, Massachusetts, United States of America; 3 Massachusetts General Hospital Cancer Center, Boston, Massachusetts, United States of America; Kaohsiung Chang Gung Memorial Hospital, TAIWAN

## Abstract

**Background:**

To find prognostic biomarkers in pretreatment dynamic contrast-enhanced MRI (DCE-MRI) water-exchange-modified (WX) kinetic parameters for advanced hepatocellular carcinoma (HCC) treated with antiangiogenic monotherapy.

**Methods:**

Twenty patients with advanced HCC underwent DCE-MRI and were subsequently treated with sunitinib. Pretreatment DCE-MRI data on advanced HCC were analyzed using five different WX kinetic models: the Tofts-Kety (WX-TK), extended TK (WX-ETK), two compartment exchange, adiabatic approximation to tissue homogeneity (WX-AATH), and distributed parameter (WX-DP) models. The total hepatic blood flow, arterial flow fraction (*γ*), arterial blood flow (*BF*_A_), portal blood flow, blood volume, mean transit time, permeability-surface area product, fractional interstitial volume (*v*_I_), extraction fraction, mean intracellular water molecule lifetime (*τ*_C_), and fractional intracellular volume (*v*_C_) were calculated. After receiver operating characteristic analysis with leave-one-out cross-validation, individual parameters for each model were assessed in terms of 1-year-survival (1YS) discrimination using Kaplan-Meier analysis, and association with overall survival (OS) using univariate Cox regression analysis with permutation testing.

**Results:**

The WX-TK-model-derived *γ* (*P* = 0.022) and *v*_I_ (*P* = 0.010), and WX-ETK-model-derived *τ*_C_ (*P* = 0.023) and *v*_C_ (*P* = 0.042) were statistically significant prognostic biomarkers for 1YS. Increase in the WX-DP-model-derived *BF*_A_ (*P* = 0.025) and decrease in the WX-TK, WX-ETK, WX-AATH, and WX-DP-model-derived *v*_C_ (*P* = 0.034, *P* = 0.038, *P* = 0.028, *P* = 0.041, respectively) were significantly associated with an increase in OS.

**Conclusions:**

The WX-ETK-model-derived *v*_C_ was an effective prognostic biomarker for advanced HCC treated with sunitinib.

## Introduction

Dynamic contrast-enhanced MRI (DCE-MRI) has a potential role in the monitoring of antiangiogenic therapy for hepatocellular carcinoma (HCC) [1–3]. Pharmacokinetic analysis of DCE-MRI data is widely applied in oncology for the measurement of vascular and tissue physiology. Extracted kinetic parameters are used for the characterization and classification of disease processes and for monitoring of treatment effects. The reliability of these measurements depends on the use of a model that accurately describes the relationship of the contrast agent (CA) concentration to the underlying tissue physiology. Because the liver receives a dual blood supply from the hepatic artery and the portal vein, dual-input tracer kinetic models hold promise for quantitative analysis of hepatic perfusion [4–7].

However, it is still unclear to what extent modeling assumptions, particularly regarding water exchange between tissue compartments, impact on parameter estimates derived from DCE-MRI data. The commonly used standard approach to DCE-MRI data analysis does not take water exchange into account, thus effectively assuming that water exchange is at the fast exchange limit (FXL) [8]. However, this assumption is physically unreasonable because deviations from the FXL condition occur when the compartmental distributions of CA and water molecules are different [9–11]. Water-exchange-modified (WX) tracer kinetic analysis of DCE-MRI data allows evaluation of intercompartmental water interchange kinetics, thus enabling additional estimation of the rate of cellular water turnover, which may play a role as a surrogate marker for quantifying the most crucial ongoing cellular metabolic turnover [12]. Therefore, it is warranted to use a model that includes water exchange effects in order to demonstrate whether water exchange has an impact on the prediction of the clinical outcome.

To date, no effort has been made to seek data comparing the relative performance of different tracer kinetic models that measure intercompartmental water exchange kinetics in DCE-MRI data regarding HCC. Our aim in this study was to find effective prognostic kinetic biomarkers of advanced HCC treated with sunitinib monotherapy for 1-year survival (1YS) and overall survival (OS), in comparison with five different WX dual-input tracer kinetic models for pretreatment liver DCE-MRI.

## Materials and Methods

### Subjects

The protocol for this phase II clinical trial on advanced HCC [1] was in compliance with Health Insurance Portability and Accountability Act regulations and was approved by the institutional review board at Dana-Farber/Harvard Cancer Center (Boston, MA). All patients were required to provide written informed consent before study participation, according to institutional and federal guidelines. All patient record/information and image data were anonymized and de-identified prior to retrospective analysis in this study. The eligibility criteria have been detailed previously [1]. The patient exclusion criteria included concurrent malignancies; significant medical comorbidities; significant cardiovascular disease, including uncontrolled hypertension, myocardial infarction, and unstable angina; New York Heart Association grade 2 or greater congestive heart failure; prolongation of QTc of more than 450 msec in screening ECG or history of familial long QT syndrome; a history of bleeding; proteinuria at baseline (more than 2 g/d); pregnancy or lactation; CNS metastases; or an inability to provide written informed consent. A total of 34 patients with histologically confirmed advanced HCC were enrolled. Fourteen of these patients were subsequently excluded because DCE-MRI data were not retrievable for this perfusion study; thus, 20 patients were included in the current study. This study cohort included 18 men and 2 women (age range, 30–79 years; mean age, 59.55 years). Full details of the clinical data of these patients have been reported in [1].

### DCE-MRI

Pretreatment DCE-MRI of the liver was performed with a 1.5 Tesla MRI system with a phased array body coil (Avanto; Siemens, New York, NY). First, a coronal T1-weighted three-dimensional (3D) volumetric interpolated breath-hold examination (VIBE) sequence of varying flip angles of 10, 15, 30, 60, and 90 degrees was performed before CA injection. Thereafter, a total of 0.1 mmol/kg bodyweight of the CA, gadolinium-diethylenetriaminepentaacetic acid (Gd-DTPA) (Magnevist; Berlex, Montville, NJ) was injected at 2 mL/sec, followed by a saline chase of 20 mL at a rate of 2 mL/sec through a 20-gauge peripheral intravenous line in the arm. A series of coronal T1-weighted 3D VIBE images was obtained after a 5-second delay following the initiation of CA injection, and the scanning was continued for up to 4 minutes and 30 seconds. The acquisition parameters were as follows: TR = 5 msec, TE = 1.58 msec, 5-mm slice thickness, 0-mm interslice gap, 20 slices, 352×384 matrix, 15-degree flip angle, and field of view of 366×400 mm. Two consecutive 7-second acquisitions were repeated 10 times with a delay of 21 seconds between them. The scanning time of every acquisition was 14 seconds with breath holding, followed by a break of 21 seconds.

### Data Processing

An image post-processing algorithm was coded in C/C++ with Visual Studio 2010 Professional Edition (Microsoft, Redmond, WA, USA). Anonymized DCE-MRI data were imported into our in-house software program and were analyzed offline by use of five different WX tracer kinetic models: the Tofts-Kety (WX-TK) model [13] based on a two-site-exchange (2SX) model for transcytolemmal exchange [14], and the extended TK (WX-ETK) model [15], two compartment exchange (WX-2CX) model [16,17], adiabatic approximation to the tissue homogeneity (WX-AATH) model [18,19], and distributed parameter (WX-DP) model [20,21] based on a three-site-two-exchange (3S2X) model for transendothelial and transcytolemmal water exchange [9] (see S1 Appendix). These models provided the following parameters: total hepatic blood flow (*BF*, mL/min/100 g), arterial flow fraction (*γ*), arterial blood flow (*BF*_A_, mL/min/100 g), portal blood flow (*BF*_PV_, mL/min/100 g), blood volume (*BV*, mL/100 g), mean transit time (*MTT*, min), capillary wall permeability-surface area product (*PS*, mL/min/100 g), fractional interstitial volume (*v*_I_), extraction fraction (*E*), mean intracellular water molecule lifetime (*τ*_C_, sec), and fractional intracellular volume (*v*_C_). A list of the various parameters and their definitions is provided in Table 1.

**Table 1:** Symbols and definitions for kinetic parameters.

Term	Definition	Unit of Measure
*C*_A_	Arterial blood concentration of tracer	g/mL
*C*_PV_	Portal blood concentration of tracer	g/mL
${\bar{C}}_{P}$	Mean concentration of tracer in the plasma compartment	g/mL
${\bar{C}}_{I}$	Mean concentration of tracer in the interstitial compartment	g/mL
*C*_T_	Concentration of tracer in tissue	g/mL
*E*_A_	Relative signal enhancement in artery	None
*E*_PV_	Relative signal enhancement in portal vein	None
*E*_T_	Relative signal enhancement in tissue	None
*R*_T_	Tissue residue function	None
*Q*_P_	Impulse response function of the plasma compartment	mL/min/mL
*Q*_I_	Impulse response function of the interstitial compartment	mL/min/mL
*Q*_T_	Impulse response function of the tissue	mL/min/mL
*F*	Total hepatic plasma flow	mL/min
*γ*	Arterial flow fraction	None
*F*_A_	Arterial plasma flow	mL/min
*F*_PV_	Portal plasma flow	mL/min
*BF*	Total hepatic blood flow	mL/min/100 g
*BF*_A_	Arterial blood flow	mL/min/100 g
*BF*_PV_	Portal blood flow	mL/min/100 g
*BV*	Blood volume	mL/100 g
*MTT*	Mean transit time	min
*PS*	Capillary wall permeability-surface area product	mL/min (or mL/min/100 g)
*PS*_C_	Cell membrane permeability-surface area product	mL/min (or mL/min/100 g)
*v*_B_	Fractional blood volume	None
*v*_P_	Fractional plasma volume	None
*v*_I_	Fractional interstitial volume	None
*v*_C_	Fractional intracellular volume	None
*E*	Extraction fraction	None
*H*_LV_	Hematocrit in major (large) vessels	None
*H*_SV_	Hematocrit in small vessels	None
*M*	Tissue mass	g
*ρ*_T_	Tissue density	g/cm^3^
*V*_P_	Volume of the plasma compartment	mL
*V*_I_	Volume of the interstitial compartment	mL
*V*_C_	Volume of the intracellular compartment	mL
*V*_T_	Tissue volume	mL
*F*/*V*_T_	Total hepatic perfusion	mL/min/mL
*F*_A_/*V*_T_	Arterial perfusion	mL/min/mL
*F*_PV_/*V*_T_	Portal perfusion	mL/min/mL
*K* ^Trans^ = *EF*/*V*_T_	Volume transfer constant between the plasma and interstitial compartments	mL/min/mL
*V*_P_/*F*	Capillary transit time	min
*V*_P_/*PS*	Capillary leakage time	min
*t*_Lag,T_	Difference in bolus arrival time between *C*_A_ (or *C*_PV_) and *C*_T_	min
*τ*_B_	Mean intravascular water molecule lifetime	sec
*τ*_I_	Mean intrainterstitial water molecule lifetime	sec
*τ*_C_	Mean intracellular water molecule lifetime	sec
*K*_BI_	Blood-to-interstitium water transfer rate	sec^-1^
*K*_IB_	Interstitium-to-blood water transfer rate	sec^-1^
*K*_CI_	Cell-to-interstitium water transfer rate	sec^-1^
*K*_IC_	Interstitium-to-cell water transfer rate	sec^-1^

For each patient, the region of interest (ROI) of a primary HCC lesion was outlined by an oncologic surgeon [K.H., with 10 years of experience in abdominal CT/MRI interpretation] in the central portions of the imaging volume for reducing inflow effects and wrap-around artifacts. Additional ROIs (mean size: 5.2 mm^2^) were placed within the aorta and the major portal vein branch for each patient, and a dual-input approach was used for analysis of the DCE-MR images [22]. The arterial input function (AIF) and the portal-venous input function (PVIF) were fitted by use of a sums-of-exponentials model [23] that describes the first-pass and recirculating inputs (see S1 Appendix). The tissue enhancement curve *E*_T_(*t*) for each voxel within the tumor ROIs was fitted separately with the five WX models. For mitigating a potential error in parameter estimation due to the discrete approximation of the continuous formula of the tissue concentration-time curve *C*_T_(*t*) [24,25], an analytic solution for *C*_T_(*t*) for each model was derived by incorporation of the AIF and PVIF models (see S1 Appendix). Model fitting was performed by use of a constrained nonlinear optimization algorithm based on MINPACK-1 [26] that yields the sum of squared residues as a measure of the goodness of fit, and allows upper and lower bounding constraints to be placed on each parameter [27].

To account for the difference in bolus arrival time between the various parts of the liver and the combined input (i.e., AIF and PVIF), an additional parameter *t*_Lag,T_ (min) was included in the expression for *C*_T_(*t*) so that an adjustable delay was imposed relative to the dual input. The mean parameter value in the tumor ROI was taken as the representative parameter value for the tumor.

### Antiangiogenic Treatment

The treatment schedule and dose modification schema have been detailed previously [1]. Briefly, patients received sunitinib at a dose of 37.5 mg daily by mouth for 4 weeks, followed by 2 weeks of rest, in 6-week cycles. Patients with grade 3 or 4 toxicities underwent dose reduction to 25 or 12.5 mg daily, respectively. Treatment was continued until progression, unacceptable toxicity, or withdrawal of consent. Patients were followed until death; one patient was alive at the end of the study, and thus the patient was censored. For this patient cohort, the median survival time was 11.42 months.

### Statistical Analysis

OS was defined as the time from the start of treatment to the date of death or of the last follow-up. Because the median survival time of patients was 11.42 months (i.e., approximately one year), and this value divides the study population into two balanced survival groups, the survival risk was estimated as one year. For each model and each kinetic parameter, the optimal parameter cut-off (threshold) value for predicting 1YS was estimated by means of receiver operating characteristic (ROC) analysis [28,29]. Because the sample size was small, a leave-one-out cross-validation (LOOCV) method, which is known to provide an unbiased estimate even in case of small samples [28,29] was used in the ROC analysis as follows: At each iteration of the LOOCV method, one of the patients was left out, and the parameter values of the remaining patients were subjected to the ROC analysis over the two survival groups of patients for generating an ROC curve. The point closest to the top-left corner on the ROC curve, i.e., the point that provides min{(1−sensitivities)^2^ + (1−specificities)^2^}, was identified as a parameter cut-off value. At the end of each iteration, the left-out patient was categorized into either the “low-risk” or “high-risk” group based on the parameter cut-off value. This process of ROC curve and cut-off value estimation was repeated at each LOOCV iteration, until all of the patients had been left out once and categorized to either the low-risk or high-risk group. Finally, after the LOOCV iterations had been completed, the cut-off value that had the highest frequency during the LOOCV iterations was identified as an optimal cut-off value for the parameter, and Kaplan-Meier analysis with log-rank test was performed for comparing the two groups generated during the LOOCV iterations.

Kaplan-Meier survival curves for patients in the low- and high-risk groups were constructed for display of the proportion of patients alive at any given time. The statistical significance in the difference between these Kaplan-Meier curves was evaluated by use of the log-rank statistic and its permutation-based significance test [29], in which 1000 random permutations were performed and the associated *P* values were calculated.

For association of a kinetic parameter with OS, univariate Cox proportional hazard regression analysis concerning the continuous parameter values was performed for testing of individual kinetic parameters for each WX kinetic model. For each parameter, *P* values were computed based on 1000 random permutations. The hazard ratio (HR) was defined as the ratio of hazards for a two-fold change in the parameter values. The HR was equal to exp(*b*), where *b* is the Cox regression coefficient. Statistical analyses were performed by use of statistical software R (version 3.0.1) and BRB-ArrayTools (version 4.4.0) [30–32].

## Results

**Fig 1:**
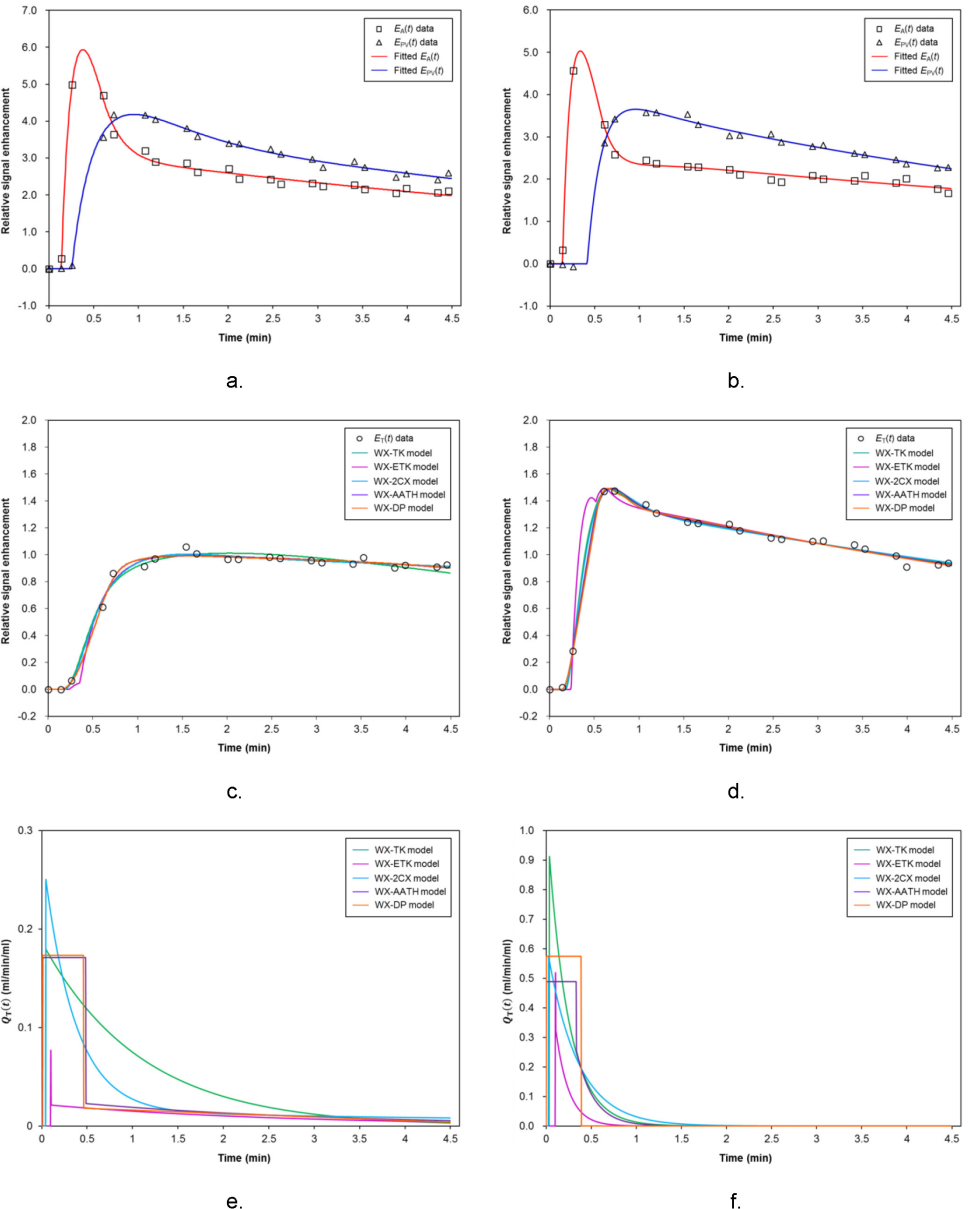
Example of fitting of arterial and portal-venous curves and tissue enhancement curves with various models. Graphs illustrate examples of (A and B) fitting of the arterial and portal input enhancement curves, (C and D) fitting of the five different models to a voxel enhancement curve which was sampled from the HCC, and (E and F) their corresponding impulse response curves (i.e., *Q*_T_(*t*) = (*F*/*V*_T_) ⋅ *R*_T_(*t*)) where two different cases are shown in the left and right columns. WX = water-exchange-modified, TK = Tofts-Kety, ETK = extended Tofts-Kety. 2CX = two compartment exchange, AATH = adiabatic approximation to tissue homogeneity, and DP = distributed parameter.

**Fig 2:**
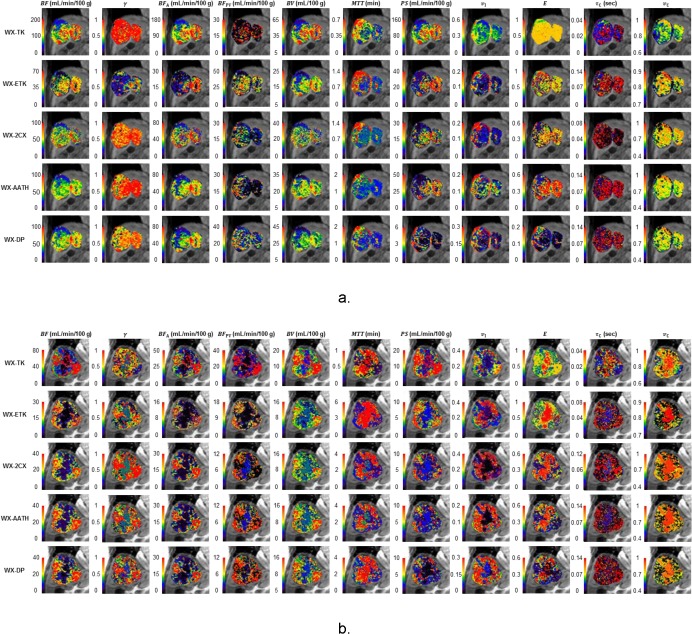
Example of kinetic parameter maps derived from various models. Parametric maps of total hepatic blood flow (*BF*), arterial flow fraction (*γ*), arterial blood flow (*BF*_A_), portal blood flow (*BF*_PV_), blood volume (*BV*), mean transit time (*MTT*), capillary wall permeability-surface area product (*PS*), fractional interstitial volume (*v*_I_), extraction fraction (*E*), mean intracellular water molecule lifetime (*τ*_C_), and fractional intracellular volume (*v*_C_) for HCC in (A) a low-risk man aged 52 years who survived for 23.87 months, and (B) a high-risk man aged 72 years who survived for 8.53 months. WX = water-exchange-modified, TK = Tofts-Kety, ETK = extended Tofts-Kety, 2CX = two compartment exchange, AATH = adiabatic approximation to tissue homogeneity, and DP = distributed parameter.

Examples of fitting of the five different WX dual-input tracer kinetic models to a voxel-level enhancement curve within HCC, the fitted *E*_T_(*t*), and the corresponding impulse response function *Q*_T_(*t*) are shown in Fig 1. Fig 2 shows two examples of cases from each of the high- and low-risk groups, with voxel-level fittings and parameter maps generated by use of the five WX models. Table 2 summarizes the mean values for the different parameters for the different WX models.

**Table 2:** Parameter values derived from the five different WX kinetic models.

Parameter	Mean ± SD for high- and low-risk populations				
	WX-TK	WX-ETK	WX-2CX	WX-AATH	WX-DP
*BF*	133.4 ± 86.17	36.74 ± 21.78	49.94 ± 26.89	49.20 ± 24.17	53.47 ± 28.13
*γ*	0.702 ± 0.255	0.459 ± 0.220	0.730 ± 0.256	0.637 ± 0.284	0.517 ± 0.263
*BF*_A_	95.36 ± 81.05	17.56 ± 17.90	38.71 ± 27.23	33.50 ± 22.58	29.44 ± 22.88
*BF*_PV_	38.09 ± 39.04	19.18 ± 13.51	11.23 ± 14.95	15.70 ± 15.34	24.03 ± 18.35
*BV*	46.10 ± 27.61	18.45 ± 9.339	17.89 ± 8.733	18.97 ± 8.092	21.01 ± 10.35
*MTT*	1.315 ± 2.066	4.605 ± 2.739	2.212 ± 1.916	1.817 ± 1.583	2.398 ± 2.768
*PS*	98.42 ± 66.30	31.63 ± 22.40	16.22 ± 10.58	20.03 ± 12.42	5.452 ± 3.649
*v*_I_	0.257 ± 0.091	0.171 ± 0.076	0.181 ± 0.108	0.163 ± 0.073	0.220 ± 0.088
*E*	0.606 ± 0.057	0.651 ± 0.104	0.346 ± 0.136	0.407 ± 0.119	0.197 ± 0.183
*τ*_C_	0.871 ± 0.684	1.050 ± 0.521	0.657 ± 0.645	1.212 ± 0.977	0.657 ± 0.627
*v*_C_	0.743 ± 0.091	0.637 ± 0.111	0.633 ± 0.139	0.639 ± 0.114	0.561 ± 0.146
*RMSE*	0.179 ± 0.126	0.152 ± 0.092	0.160 ± 0.108	0.151 ± 0.101	0.168 ± 0.128

Note.—SD = standard deviation, WX = water-exchange-modified, TK = Tofts-Kety, ETK = extended Tofts-Kety, 2CX = two compartment exchange, AATH = adiabatic approximation to the tissue homogeneity, DP = distributed parameter, *BF* = total hepatic blood flow (in mL/min/100 g), *γ* = arterial flow fraction (unitless), *BF*_A_ = arterial blood flow (in mL/min/100 g), *BF*_PV_ = portal blood flow (in mL/min/100 g), *BV* = blood volume (in mL/100 g), *MTT* = mean transit time (in min), *PS* = capillary wall permeability-surface area product (in mL/min/100 g), *v*_I_ = fractional interstitial volume (unitless), *E* = extraction fraction (unitless), *τ*_C_ = mean intracellular water molecule lifetime (sec), *v*_C_ = fractional intracellular volume (unitless), and *RMSE* = root-mean-square error between original and fitted *E*_T_(*t*).

### One-year Survival

**Fig 3:**
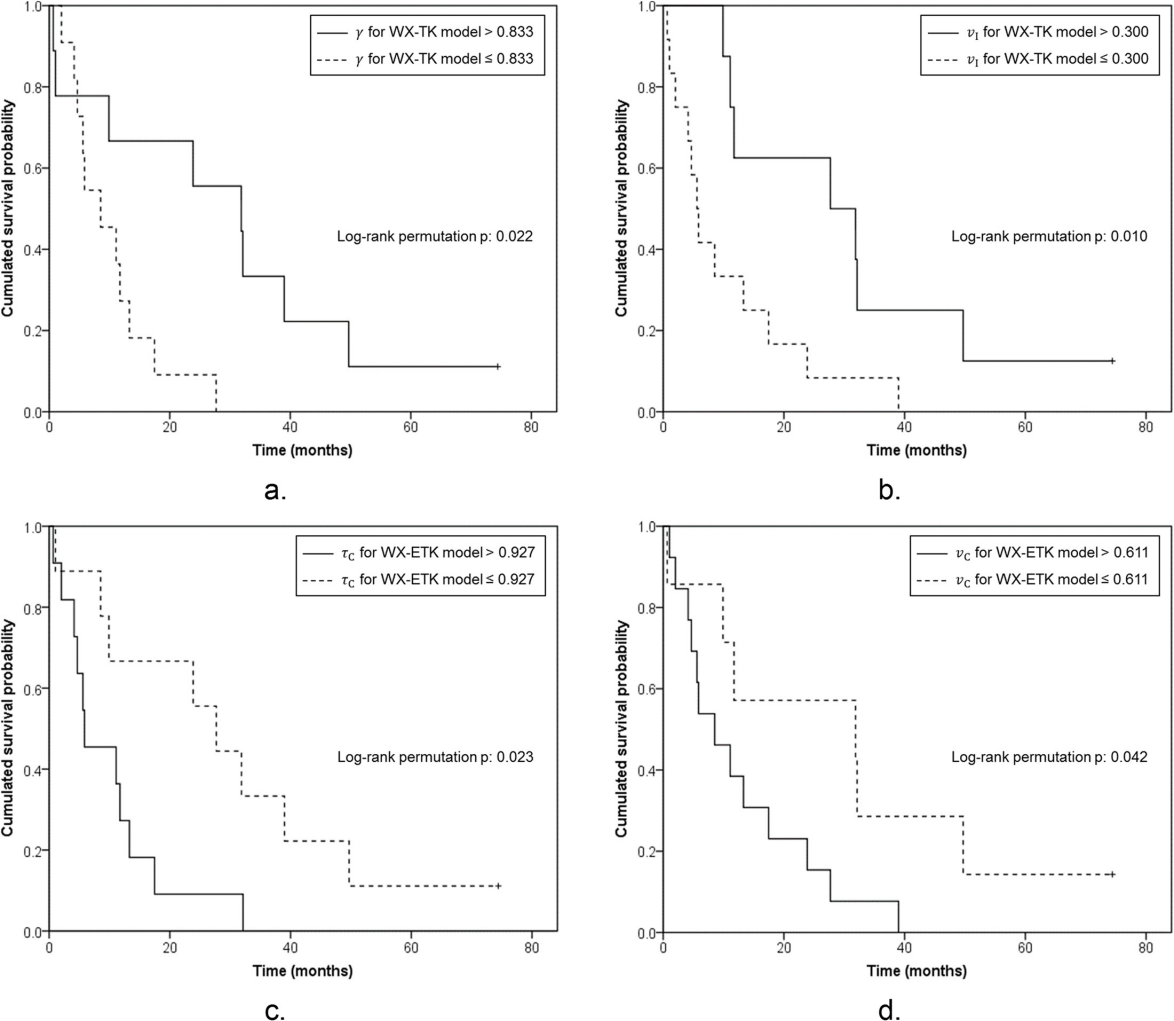
Kaplan-Meier curves for kinetic parameters predictive of 1-year survival. Cross-validated Kaplan-Meier plots for (A) arterial flow fraction (*γ*) and (B) fractional interstitial volume (*v*_I_) derived from the water-exchange-modified Tofts-Kety (WX-TK) model, and (C) mean intracellular water molecule lifetime (*τ*_C_) and (D) fractional intracellular volume (*v*_C_) derived from the water-exchange-modified extended Tofts-Kety (WX-ETK) model. Survival of patients with advanced HCC treated with sunitinib was better with *γ* over 0.833 and *v*_I_ over 0.300 in the WX-TK model, and with *τ*_C_ at most 0.927 sec and *v*_C_ at most 0.611 in the WX-ETK model.

Table 3 shows the optimized cut-off values of the parameters determined by ROC analysis with LOOCV for each model along with the *P* values for the log-rank permutation test. For the WX-2CX, WX-AATH and WX-DP model, the cross-validated Kaplan-Meier curves were not significantly different between the two groups (*P*>0.05). Only the WX-TK-model-derived *γ* and *v*_I_, and the WX-ETK-model-derived *τ*_C_ and *v*_C_ were significant biomarkers for 1YS (Fig 3). The WX-TK-model-derived *γ* and *v*_I_ were both lower in the high-risk than in the low-risk group, with cut-off values of 0.733 (*P* = 0.022) and 0.300 (*P* = 0.010), respectively. The WX-ETK-model-derived *τ*_C_ and *v*_C_ were both higher in the high-risk than in the low-risk group, with cut-off values of 0.927 sec (*P* = 0.023) and 0.611 (*P* = 0.042), respectively.

**Table 3:** Optimal cut-off values of parameters and their log-rank test results from leave-one-out cross-validated Kaplan-Meier analysis in terms of 1-year survival.

Parameter	Cut-off value (*P*-value)				
	WX-TK	WX-ETK	WX-2CX	WX-AATH	WX-DP
*BF*	111.5 (0.345)	36.66 (0.315)	56.04 (0.220)	56.23 (0.315)	59.22 (0.682)
*γ*	0.833 (**0.022**)*	0.475 (0.871)	0.763 (0.277)	0.591 (0.338)	0.572 (0.080)
*BF*_A_	47.19 (0.172)	12.09 (0.359)	39.18 (0.662)	36.42 (0.952)	28.58 (0.662)
*BF*_PV_	10.45 (0.776)	23.99 (0.447)	5.592 (0.233)	10.36 (0.795)	15.03 (0.491)
*BV*	37.27 (0.345)	16.89 (0.315)	20.66 (0.379)	21.30 (0.168)	18.69 (0.634)
*MTT*	0.380 (0.442)	5.575 (0.597)	1.709 (0.341)	1.333 (0.163)	1.414 (0.163)
*PS*	80.52 (0.504)	26.31 (0.315)	19.95 (0.725)	20.04 (0.644)	2.697 (0.782)
*v*_I_	0.300 (**0.010**)*	0.123 (0.161)	0.207 (0.344)	0.142 (0.206)	0.209 (0.460)
*E*	0.603 (0.682)	0.635 (0.981)	0.298 (0.367)	0.413 (0.224)	0.118 (0.377)
*τ*_C_	0.482 (0.199)	0.927 (**0.023**)*	0.376 (0.831)	0.819 (0.588)	0.284 (0.702)
*v*_C_	0.728 (0.131)	0.611 (**0.042**)*	0.533 (0.282)	0.646 (0.468)	0.518 (0.194)

Note.—WX = water-exchange-modified, TK = Tofts-Kety, ETK = extended Tofts-Kety, 2CX = two compartment exchange, AATH = adiabatic approximation to the tissue homogeneity, DP = distributed parameter, *BF* = total hepatic blood flow (in mL/min/100 g), *γ* = arterial flow fraction (unitless), *BF*_A_ = arterial blood flow (in mL/min/100 g), *BF*_PV_ = portal blood flow (in mL/min/100 g), *BV* = blood volume (in mL/100 g), *MTT* = mean transit time (in min), *PS* = capillary wall permeability-surface area product (in mL/min/100 g), *v*_I_ = fractional interstitial volume (unitless), *E* = extraction fraction (unitless), *τ*_C_ = mean intracellular water molecule lifetime (sec), and *v*_C_ = fractional intracellular volume (unitless). Bold numbers with asterisk (*) indicate a statistically significant difference in the 1000 log-rank permutation test (two-sided *P*<0.05).

### Overall Survival

Table 4 shows the hazard ratios and corresponding *P* values for the parameters determined by the univariate Cox proportional hazard model for each kinetic model. The results showed that the WX-TK (HR = 21.51, *P* = 0.034), WX-ETK (HR = 8.418, *P* = 0.038), WX-AATH (HR = 10.14, *P* = 0.028), and WX-DP-model-derived *v*_C_ (HR = 4.213, *P* = 0.041), and the WX-DP-model-derived *BF*_A_ (HR = 0.716, *P* = 0.025), were associated with OS, indicating that the risk of death rises by approximately 35.9%, 23.7%, 26.1%, and 15.5% for an increase of 0.1 (10%) in the WX-TK, WX-ETK, WX-AATH, and WX-DP-model-derived *v*_C_, respectively, and falls by 28.4% for an increase of 1 mL/min/100 g in the WX-DP-model-derived *BF*_A_.

**Table 4:** Results of univariate Cox’s proportional hazards regression analysis of parameters in terms of overall survival.

Parameter	Hazard ratio (*P*-value)				
	WX-TK	WX-ETK	WX-2CX	WX-AATH	WX-DP
*BF*	0.785 (0.217)	0.747 (0.138)	0.717 (0.076)	0.680 (0.092)	0.693 (0.092)
*γ*	0.879 (0.562)	0.961 (0.890)	0.927 (0.747)	0.843 (0.376)	0.792 (0.198)
*BF*_A_	0.753 (0.063)	0.796 (0.203)	0.757 (0.093)	0.734 (0.051)	0.716 (**0.025**)*
*BF*_PV_	1.183 (0.248)	0.930 (0.564)	1.012 (0.914)	1.049 (0.760)	1.076 (0.684)
*BV*	0.805 (0.362)	0.687 (0.218)	0.660 (0.055)	0.557 (0.069)	0.642 (0.110)
*MTT*	1.247 (0.166)	1.125 (0.695)	1.356 (0.165)	1.371 (0.180)	1.275 (0.266)
*PS*	0.816 (0.272)	0.815 (0.307)	0.756 (0.173)	0.747 (0.195)	0.959 (0.829)
*v*_I_	0.438 (0.053)	0.660 (0.307)	0.942 (0.788)	0.660 (0.332)	0.577 (0.196)
*E*	1.975 (0.804)	1.656 (0.612)	1.331 (0.541)	1.322 (0.694)	1.181 (0.373)
*τ*_C_	0.821 (0.424)	1.353 (0.415)	1.237 (0.326)	1.031 (0.883)	1.053 (0.828)
*v*_C_	21.51 (**0.034**)*	8.418 (**0.038**)*	4.109 (0.094)	10.14 (**0.028**)*	4.213 (**0.041**)*

Note.—WX = water-exchange-modified, TK = Tofts-Kety, ETK = extended Tofts-Kety, 2CX = two compartment exchange, AATH = adiabatic approximation to the tissue homogeneity, DP = distributed parameter, *BF* = total hepatic blood flow (in mL/min/100 g), *γ* = arterial flow fraction (unitless), *BF*_A_ = arterial blood flow (in mL/min/100 g), *BF*_PV_ = portal blood flow (in mL/min/100 g), *BV* = blood volume (in mL/100 g), *MTT* = mean transit time (in min), *PS* = capillary wall permeability-surface area product (in mL/min/100 g), *v*_I_ = fractional interstitial volume (unitless), *E* = extraction fraction (unitless), *τ*_C_ = mean intracellular water molecule lifetime (sec), and *v*_C_ = fractional intracellular volume (unitless). Bold numbers with asterisk (*) indicate a statistically significant difference in the 1000 permutation test for hazard ratio in univariate Cox proportional hazards analysis (two-sided *P*<0.05).

Overall, the WX-ETK-model-derived *v*_C_ was not only identified as a statistically significant prognostic biomarker for 1YS, but was also statistically significantly associated with OS.

## Discussion

Several studies have assessed the impact of water exchange on the estimation of pharmacokinetic parameters, but no definite conclusions have been reached about the significance of water exchange effects in clinical practice. Bains *et al*. analyzed DCE-MRI data by using the WX-2CX model under the FXL and no-exchange-limit conditions, and they compared kinetic parameters derived from DCE-MRI with those obtained from exchange-insensitive DCE-CT in bladder cancer [33]. They concluded that the cell-to-interstitium water transfer rate (*K*_CI_) has a negligible effect on estimates of kinetic parameters, whereas the blood-to-interstitium water transfer rate (*K*_BI_) influences estimates of the fractional plasma volume (*v*_P_). Huang *et al*. compared the FXL standard and shutter-speed (fast-exchange regime) approaches of the TK model, and they demonstrated that relieving of the FXL constraint leads to a higher performance for breast cancer diagnosis [11]. Using computer simulations in the WX-ETK model, Paudyal *et al*. demonstrated that the effect of a variation of *K*_BI_ is significant for *v*_P_ and is minimal for the transfer constant (*K* ^Trans^ = *EF*/*V*_T_) and the fractional interstitial volume (*v*_I_) when *K*_CI_ is held constant, whereas *K* ^Trans^ and *v*_I_ are influenced by a variation in *K*_CI_ when *K*_BI_ is held constant [34]. Using simulated DCE-MRI data, Zhang and Kim assessed four different kinetic models (FXL standard ETK, shutter-speed ETK, FXL standard AATH, and WX-AATH models), and they found that *K* ^Trans^, *v*_I_, and *v*_P_ values estimated from the WX-AATH model had the highest accuracy [35]. Because the rate of water exchange is related to the cell type, size, shape, and membrane permeability [10], the vascular-interstitial and cellular-interstitial water exchange can vary significantly between normal and tumor tissue [36]. Therefore, DCE-MRI yields a variety of water exchange regimes, and here we focused on a water exchange paradigm based on a full 2SX model [14] or a full 3S2X model [34] that can fully describe the effects from fast to intermediate or slow water exchange (see S1 Appendix). To the authors’ knowledge, this is the first comparative study that identified a prognostic WX tracer kinetic model and parameter for 1YS and OS of patients with advanced HCC who received sunitinib monotherapy.

Our results showed that the WX-ETK-model-derived *v*_C_ was identified as the most effective prognostic biomarker for OS and 1YS, which was higher in the high-risk than in the low-risk group. We assume that these results are associated with tumor hypoxia. Although HCC is a highly angiogenic cancer, it is also characterized by hypoxia that promotes HCC growth, progression, angiogenesis, and resistance to therapies [37]. Thus, the high-risk patients may have relatively more hypoxic HCC [38]. In a hypoxic environment, the mitochondrial oxygen consumption rate and adenosine triphosphate (ATP) production are reduced, which hinders inter alia active transport into tumor cells [39]. The reduction of ATP by a decreased oxygen supply causes disturbances in membrane ion translocation that result in cell swelling, which may induce high *v*_C_ [40]. On the other hand, Heider et al. [41] demonstrated that the tumor blood flow was correlated positively with tumor oxygenation in a CT perfusion study of cervical cancer. Thus, it is assumed that low tumor *BF*_A_ is associated with a hypoxic tumor microenvironment, which leads to poor-outcome-promoting oncogenic mutations, cell survival, and more aggressive behavior of tumors [42]. Besides, low tumor *BF*_A_, which leads the disturbed microcirculation of antiangiogenic agents and the deterioration of their diffusion conditions, may result in poor OS of HCC patients treated with sunitinib [12,39,43].

Our results also showed that the parameters that were found to be statistically significant prognostic biomarkers for 1YS (the WX-TK-model-derived *γ* and *v*_I_) were not necessarily statistically significantly associated with OS. In contrast, the WX-DP-model-derived *BF*_A_, and the WX-TK, WX-AATH and WX-DP-model-derived *v*_C_ were not statistically significant prognostic biomarkers for 1YS, although they were statistically significantly associated with OS. The time scale for events can be classified as either continuous or discrete, and the methods applied to one type of time scale do not necessarily apply to the other [44]. A fundamental problem with methods based on dividing the timeline into discrete intervals is the loss of information. Therefore, our results suggest that analyzing both continuous- and discrete-time events may be necessary for finding a robust prognostic biomarker.

As antiangiogenic agents might exert different pharmacokinetic effects on tumor flow and permeability, a method that can separately estimate the plasma flow *F* (in mL/min) and *PS* may be of central importance for understanding tissue hemodynamics. The early version of the TK model, which has been used most frequently, facilitates the extraction of the volume transfer constant *K* ^Trans^ (in min^-1^), the physiologic interpretation of which reflects a combination of *F* and *PS* [15], but it has been known that their separate estimates are impossible with the TK model. The relative magnitude of *PS* and *F* determines *E*, i.e., the fraction of the mass of CA arriving at the tissue which leaks into the interstitial space in a single passage through the vasculature.

Under the mixed flow- and permeability-limited condition, *K* ^Trans^ = *EF*/*V*_T_ [13,15], where *V*_T_ is the examined tissue volume (in mL), and *F*/*V*_T_ is the tissue perfusion (in mL/min/mL). With further assumption that *v*_P_ ≪ *v*_I_, a single compartment TK model can be derived (see S1 Appendix). The original TK model additionally presumed that CA in the plasma compartment can be neglected (i.e., *v*_P_ ≅ 0) [45]. However, a separate determination of *E* and *F*/*V*_T_ is impossible with the assumption that *v*_P_ ≅ 0. In this study, we refined the TK model parameterization so that *K* ^Trans^ was decomposed into *E* and *F*/*V*_T_, by assuming that *v*_P_ ≪ *v*_I_. The assumption of *v*_P_ ≪ *v*_I_ leads to *C*_T_(*t*) ≅ *v*_I_ *C*_I_(*t*), where *C*_I_(*t*) is the CA concentration in the interstitial compartment, and thereby the capillary transit time *V*_P_/*F* (in min) and the capillary leakage time *V*_P_/*PS* (in min) are negligible, where *V*_P_ = *v*_P_ *V*_T_ is the plasma volume (in mL). However, their reciprocals, *F*/*V*_P_ (in mL/min/mL) and *PS*/*V*_P_ (in mL/min/mL), are both infinite if *v*_P_ ≅ 0. Because *F* and *PS* cannot be calculated without *v*_P_ (i.e., *E* becomes undefined), the assumption that *v*_P_ ≅ 0 may contradict with the original TK model. On the other hand, if *v*_P_ ≠ 0, then *F*/*V*_P_ and *PS*/*V*_P_ are finite, and thus *F*/*V*_T_ = *v*_P_ *F*/*V*_P_ = *v*_I_ *F*/*V*_I_ and *PS*/*V*_T_ = *v*_P_ *PS*/*V*_P_ = *v*_I_ *PS*/*V*_I_, where *V*_I_ = *v*_I_ *V*_T_ = (*v*_I_/*v*_P_)*V*_P_ is the interstitial volume (in mL), are not only calculable, but also *E* can be defined (e.g., $E=1-e^{-\left(PS/V_{\text{P}}\right)/\left(F/V_{\text{P}}\right)}$). As a result, *F* and *PS* can be measured separately with our new parameterization rules of the TK model. These parameterization rules, which have originally been proposed by Brix *et al*. for evaluation of the 2CX model [16], apply to all of the kinetic models considered in this study (see S1 Appendix), and the enable fair comparisons among different kinetic models. Even if parameterization rules are different between the original approaches and our methods on tracer kinetic modeling, the mass balance principle of CA in the capillary-tissue system is identical for them.

To date, very few data have been reported on the issue of limited vascular-interstitial water exchange and its effect on DCE-MRI. Here, we assumed that the vascular-interstitial exchange behavior would virtually be identical between the CA (Gd-DTPA) and water molecules (see S1 Appendix). In the absence of the CA, water exchange is usually regarded as being in the FXL [8,14], but in its presence, it enters the interstitium from the plasma and increases the relaxation rate of interstitial water while the T1 of water in the cell remains the same, which may lead to significant transient sorties away from the precontrast water exchange state. Even though water molecules are much lighter (≅18 g/mol) than Gd-DTPA molecules (Magnevist, 938 g/mol), and the self-diffusion coefficient of water (*D* = 2.2 × 10^−9^ m^2^/sec) is higher than that of Gd-DTPA (Magnevist, *D* = 2.6 × 10^−10^ m^2^/sec) [46], it should be noted that slow or fast water exchange does not refer directly to the speed with which the water molecule moves between the two spaces, but to the ratio of this motion to the difference in relaxation rates of the two spaces [47]. If the two spaces have identical relaxation rates, they will always be described as being in the FXL but slowly the water molecules exchange between them, because no distinction can be made between their relaxation properties. Contrarily, if the two spaces have an order-of-magnitude difference in their each relaxation rates, they will be in the slow exchange regime even though water moves very rapidly between them, because their relaxation properties will always be the major focus of the decision of water exchange [48].

The addition of CA to the interstitial space does not change the motion of water molecules, but it increases the intrinsic relaxation rate of the interstitial space. Given the large difference in relaxation rates in the two spaces immediately after the CA injection, the water exchange would significantly depart from the FXL, because the CA might not have had time to pass into the interstitial space while being present in high concentration in the intravascular space. For example, with an intact blood-brain barrier that has very low permeability, the vascular-interstitial water exchange slowly approaches the limit during the first pass of a bolus of Gd-DTPA [49]. On the other hand, if the CA enters the interstitial space quickly, the effect of slow water exchange is short-lived. For example, in the heart, where the first-pass extraction of Gd-DTPA is significant, the slow-water-exchange effect decreases quickly [49]. As such, the vascular-interstitial water exchange with typical CA concentrations is in the slow- to intermediate-exchange regime [8], and the trends in water mobility to the difference in relaxation rates of the two spaces seem to be more like Gd-DTPA exchange behavior rather than the inherent speed of movement of the water. Furthermore, even if the vascular-interstitial water exchange rates may appear to be faster in tumors where the vascular permeability is larger, they are probably dependent on the geometric and hemodynamic heterogeneity of microvascular networks as well as the microvascular permeability.

HCC is a heterogeneous disease with multiple etiologies that has an abnormal blood flow and is excessively leaky [38]. Setting the water exchange rate to a value reported in the literature is unlikely to reflect a complex phenomenon involving multiple exchange behaviors in the context of a spatially heterogeneous tumor microenvironment. As an alternative, model fitting can be done by considering the mean intravascular water molecule lifetime *τ*_B_ as an additional free parameter independent from the exchange rate of CA [9]. However, the accuracy and precision in such an approach may be low [35]. The reason for this is not obvious, but one could hypothesize that the estimation of *τ*_B_ might be difficult without the aid of tracer kinetic parameters because *τ*_B_ is calculated based on the ratio of *BV* to *PS* [8]. In contrast, *τ*_C_ can be used as an independent variable [14], because Gd-DTPA does not permeate the cell membrane and pass into the intracellular space, whereas the cellular-interstitial water exchange is intermediate to fast [8]. Therefore, although the speed of intercompartmental water exchange cannot be set to a single value, our approach would be a plausible implicit compromise for the evaluation of tumor angiogenesis.

When more complex models such as WX kinetic models are used for data analysis, the goodness of fit alone may not be sufficient to ensure that the estimated parameters truly reflect the corresponding biophysical properties of the tissue [35]. Therefore, in this study, we compared the prognostic ability for patient survival among different WX kinetic models under the same experimental conditions to identify clinically relevant biomarkers. Although such biomarkers may provide clinical realism and biologic validity, the precision in the parameter estimates may be influenced by the low temporal resolution of the DCE-MRI data. In addition, because the data-fitting process is formulated as an optimization task, the nonlinear characteristics of the kinetic model may lead to a convergence problem. Moreover, as DCE-MRI data are obtained sequentially at fixed time points, issues of discontinuity may arise which are caused by the initial step of the impulse residue function at the bolus arrival time (any model) and at the capillary transit time (AATH and DP models) [25]. To mitigate these problems, we first imposed upper and lower boundary constraints to the parameter values in order to avoid its over- and under-fitting. Second, we employed standard gradient-based optimization algorithms with an explicit analytic solution via continuous-time-domain convolution between the hepatic dual-input function (AIF and PVIF) and *Q*_T_(*t*) for each kinetic model, in which the WX dual-input kinetic models were extended include the primary (first-pass) and recirculation bolus arrival times as a free continuous parameter *t*_Lag,T_ (see S1 Appendix).

It should also be noted that both the AIF and PVIF hold the same smooth and integrable functional forms, so that their scaling constants and bolus arrival times constrain kinetic parameter values at any temporal resolution. Therefore, the occurrences of discontinuities in the impulse residue function do not necessarily result in a discontinuous or nondifferentiable *C*_T_(*t*), which poses difficulties for curve-fitting algorithms employing gradient-based optimization schemes. Although some parameters such as *BF*, *BV*, and *t*_Lag,T_ might be influenced by the low temporal resolution of the DCE-MRI data, the continuous-time analytic formulations of *C*_T_(*t*) would lower the potential bias in parameter estimates [25]. A more precise analysis may require a higher temporal resolution. Investigation of the impact of modifications in DCE-MRI acquisition techniques on kinetic parameters was beyond the scope of this study, although it is an important issue that deserves more thorough investigation in future studies.

Accurate estimates of *v*_P_ is clinically useful for assessing the integrity of the tumor microvasculature [50,51] and for monitoring the response to antiangiogenic therapy [52,53]. Although *BV*, in which the ratio *BV*/*v*_P_ is a constant, was not chosen as a prognostic biomarker in this study, *v*_P_ might have an influence on estimation of *v*_C_. The FXL assumption may bias the accuracy and reproducibility of *v*_P_ measurements because of the restricted vascular-interstitial water exchange [54]. In addition, if the concentration of CA in the interstitial space were to reach a significant level, the cellular-interstitial water exchange would depart from the FXL [10,14,55]. Because the mixing phase, during which the injected bolus is mixed with the blood plasma and interstitial compartments, lasts up to about 2 minutes [45], the effects of slow cellular-interstitial water exchange would be felt during the longer total measurement time (4.5 minutes) in this study. The full 3S2X model determines kinetic parameters, including both the vascular-interstitial and cellular-interstitial water transfer rates, but the full 2SX model includes only the cellular-interstitial water transfer rate [9]. Therefore, the fact that the full 3S2X model (WX-ETK model) outperformed the full 2SX model (WX-TK model) in this study confirms that the inclusion of the vascular-interstitial water exchange is necessary for the effective risk assessment of tumor characteristics such as vascularity, cellularity, and hypoxia.

There are limitations to our study. First, this is a retrospective analysis on a relatively small number of patients from a Phase II clinical trial, although it serves as a pilot comparative study. Second, the single-arm study design of the clinical trial did not allow us to evaluate the predictive value of the kinetic parameters as imaging biomarkers; instead, the prognostic value of the kinetic parameters was evaluated by means of statistical resampling methods. Third, the prognostic value was evaluated on the kinetic parameters only on the pretreatment DCE-MRI, because the primarily clinical goal of this study was to find a prognostic imaging biomarker that estimates the prognosis of patients before the treatment starts. Evaluation of the prognostic value of the kinetic parameters of the post-treatment DCE-MRI, which may have an effect on the 1YS and OS, remains for further study. Forth, survival risk estimation based on the selected cut-off values of the kinetic parameters with use of ROC analysis may result in an overestimate of the prognostic value of the parameters. Ideally, this type of analysis should have been performed based on large independent training and testing data sets. When the data size is not large enough, cross-validation methods are known to be more efficient than splitting of the data set into training and testing subsets [29]. We thus performed LOOCV and permutation tests to obtain an estimate of the prognostic value of the kinetic parameters. We believe that we were able to estimate a reasonably unbiased and generalizable prognostic value of the kinetic parameters with respect to 1YS as well as association with OS. Finally, the clinical value of the prognostic imaging biomarker found in this study may be limited to the patients who are treated with sunitinib monotherapy. Currently, sunitinib monotherapy is not an accepted treatment for patients with advanced HCC in clinical practice. Thus, the clinical value of the prognostic imaging biomarker for the patients treated with sorafenib, the current standard treatment, and other antiangiogenic therapies remains unknown. We believe, however, that the findings on the prognostic value of the kinetic parameters from DCE-MRI data have significant implications for antiangiogenic agents, and thus warrant further validation in larger prospective studies.

## Conclusion

In conclusion, kinetic parameters derived from WX dual-input tracer kinetic models of DCE-MRI data can be effective prognostic biomarkers for survival of patients with advanced HCC. The choice of kinetic models influences the predictability of survival, and the WX-ETK-model-derived *v*_C_ was found to be an effective prognostic biomarker for both 1YS and OS in patients with advanced HCC who received sunitinib monotherapy. The fact that the *v*_C_ biomarker is available only from the WX kinetic formulation indicates that the water transport properties of cells is an important predictor of cancer cell development.

## Supporting Information

S1 Appendix Water-exchange-modified tracer kinetic modeling in DCE-MRI. (DOCX)
