# Supplementary material for: Water-Exchange-Modified Kinetic Parameters from Dynamic Contrast-Enhanced MRI as Prognostic Biomarkers of Survival in Advanced Hepatocellular Carcinoma Treated with Antiangiogenic Monotherapy
Source: PLoS One. 2015 Sep 14;10(9):e0136725. doi: 10.1371/journal.pone.0136725 (PMC4569468; doi:10.1371/journal.pone.0136725)
Supplement: S1 Appendix — (DOCX) [file pone.0136725.s001.docx]

$BF$ $\gamma$ ${BF}_{A}$ ${BF}_{PV}$ $BV$ $MTT$ $PS$ $v_{I}$ $E$ $\tau_{C}$ $v_{C}$

Water-exchange-modified Tracer Kinetic Modeling in Dynamic Contrast-enhanced MRI

This appendix provides readers with uniform notations and definitions of the kinetic parameters across the five WX dual-input tracer kinetic models (WX-TK, WX-ETK, WX-2CX, WX-AATH, and WX-DP) used in this study, along with the fundamental biophysical concepts and tracer kinetic principles of dynamic contrast-enhanced imaging, based on which these models were developed. These WX models account for the effects of cellular-interstitial (WX-TK) or vascular-interstitial-cellular water exchange (WX-ETK, WX-2CX, WX-AATH, and WX-DP) on standard models (TK, ETK, 2CX, AATH, and DP) that use FXL, neglecting water exchange effects.

Each of the five tracer kinetic models is a single (TK) or a two (ETK, 2CX, AATH, and DP) compartmental system that can be described by a pair of linear differential equations that represent *conservation of tracer mass*. The five models differ in the formulation of these differential equations due to their different assumptions. The formal solution for each model can be obtained by solving of the simultaneous paired differential equations. For incorporating water exchange with each kinetic model, an intracellular pool is added as a third compartment, where the cellular-interstitial water exchange is described by the mean intracellular water molecule lifetime $\tau_{C}$. The effect of water exchange on estimates of kinetic parameters is assessed based on a full 2SX model (WX-TK) [1,2] or a full 3S2X model (WX-ETK, WX-2CX, WX-AATH, and WX-DP) [3-5].

Because the vascular-interstitial water exchange is related to the capillary wall permeability-surface area product ($PS$) or extraction-flow product ($EF$) [6,7], use of a fixed or predetermined water exchange rate constant assumes a limited value for $PS$ (or $EF$) [8]. As an alternative, we assume that the vascular-interstitial (transendothelial) water exchange behavior is much like the CA exchange behavior between the plasma and interstitial compartments. Therefore, the dependence of the vascular-interstitial water exchange on $PS$ (or $EF$) and that of the cellular-interstitial (transcytolemmal) water exchange on $\tau_{C}$ are specified mathematically; then they are constrained during fitting of each kinetic model.

The liver receives blood from the hepatic artery and the portal vein. Thus, the net input function is modeled as a weighted sum of dual-input functions; $\gamma C_{A}\left( t \right)+\left( 1-\gamma\right)C_{\mathrm{PV}}\left( t \right)$, where $\gamma$, $C_{A}\left( t \right)$, and $C_{\mathrm{PV}}\left( t \right)$ are kinetic parameters as defined in Table 1. Each of the input functions is further decomposed into $C_{A}\left( t \right)=C_{B,A}\left( t \right)+C_{B,A}\left( t \right)\bigotimes G_{A}\left( t \right)$ and $C_{\mathrm{PV}}\left( t \right)=C_{B,PV}\left( t \right)+C_{B,PV}\left( t \right)\bigotimes G_{\mathrm{PV}}\left( t \right)$, where $C_{B,A}\left( t \right)=a_{B,A}te^{-\mu_{B,A}t}$ and $C_{B,PV}\left( t \right)=a_{B,PV}te^{-\mu_{B,PV}t}$ describe the first pass of the bolus for the arterial and portal venous components, and $G_{A}\left( t \right)=a_{G,A}e^{-\mu_{G,A}t}$ and $G_{\mathrm{PV}}\left( t \right)=a_{G,PV}e^{-\mu_{G,PV}t}$ are the body transfer function (BTF) that models leakage into the whole-body interstitial space. The convolutions are analytically tractable, and both $C_{A}\left( t \right)$ and $C_{\mathrm{PV}}\left( t \right)$ are the superposition of the bolus shape (first pass) and its shape after modification by the BTF (recirculation), representing a sum-of-exponentials function [9]. By imposing the time lag of the first-pass bolus arrival to the hepatic artery and the portal vein ($t_{Lag,A1}$ and $t_{Lag,PV1}$), and that of the first pass to the recirculation onset ($t_{Lag,A2}$ and $t_{Lag,PV2}$), $C_{A}\left( t \right)$ and $C_{\mathrm{PV}}\left( t \right)$ are represented by

$C_{A}\left( t \right)=a_{B,A}\left( t-t_{Lag,A1} \right)e^{-\mu_{B,A}\left( t-t_{Lag,A1} \right)}u\left( t-t_{Lag,A1} \right)-\frac{a_{B,A}a_{G,A}}{\mu_{B,A}-\mu_{G,A}}\left\{ \left( t-t_{Lag,A1}-t_{Lag,A2} \right)e^{-\mu_{B,A}\left( t-t_{Lag,A1}-t_{Lag,A2} \right)}-\frac{e^{-\mu_{G,A}\left( t-t_{Lag,A1}-t_{Lag,A2} \right)}-e^{-\mu_{B,A}\left( t-t_{Lag,A1}-t_{Lag,A2} \right)}}{\mu_{B,A}-\mu_{G,A}} \right\}u\left( t-t_{Lag,A1}-t_{Lag,A2} \right),$

(1)


and

$$C_{PV}\left( t \right)=a_{B,PV}\left( t-t_{Lag,PV1} \right)e^{-\mu_{B,PV}\left( t-t_{Lag,PV1} \right)}u\left( t-t_{Lag,PV1} \right)-\frac{a_{B,PV}a_{G,PV}}{\mu_{B,PV}-\mu_{G,PV}}\left\{ \left( t-t_{Lag,PV1}-t_{Lag,PV2} \right)e^{-\mu_{B,PV}\left( t-t_{Lag,PV1}-t_{Lag,PV2} \right)}-\frac{e^{-\mu_{G,PV}\left( t-t_{Lag,PV1}-t_{Lag,PV2} \right)}-e^{-\mu_{B,PV}\left( t-t_{Lag,PV1}-t_{Lag,PV2} \right)}}{\mu_{B,PV}-\mu_{G,PV}} \right\}u\left( t-t_{Lag,PV1}-t_{Lag,PV2} \right),$$

(2)


where $u\left( t \right)$ is the unit step function.

We consider dual-input sources of the plasma flow $F$ to the liver, that is, arterial plasma flow from the hepatic artery, $F_{A}$, and portal plasma flow from the portal vein, $F_{\mathrm{PV}}$. Because these inputs join in the capillary bed, they can be effectively replaced by a single input with mixed arterial and portal contributions. Assuming that the arterial and portal blood concentrations, $C_{A}\left( t \right)$ and $C_{\mathrm{PV}}\left( t \right)$, can be obtained from DCE-MR images, the concentration for the liver tissue, $C_{T}\left( t \right)$, can be expressed as follows [10,11]:

$$C_{T}\left( t \right)=R_{T}\left( t-t_{Lag,T} \right)\bigotimes\frac{\left( {F_{A}}/{V_{T}} \right)C_{A}\left( t \right)+\left( {F_{PV}}/{V_{T}} \right)C_{PV}\left( t \right)}{1-H_{LV}}=\frac{F}{V_{T}}R_{T}\left( t-t_{Lag,T} \right)\bigotimes\frac{\gamma C_{A}\left( t \right)+\left( 1-\gamma\right)C_{PV}\left( t \right)}{1-H_{LV}}=Q_{T}\left( t-t_{Lag,T} \right)\bigotimes\frac{\gamma C_{A}\left( t \right)+\left( 1-\gamma\right)C_{PV}\left( t \right)}{1-H_{LV}}=\left\{ v_{P}Q_{P}\left( t-t_{Lag,T} \right)+v_{I}Q_{I}\left( t-t_{Lag,T} \right) \right\}\bigotimes\frac{\gamma C_{A}\left( t \right)+\left( 1-\gamma\right)C_{PV}\left( t \right)}{1-H_{LV}}=v_{P}\bar{C}_{P}\left( t \right)+v_{I}\bar{C}_{I}\left( t \right),$$

(3)


where $H_{\mathrm{LV}}$ is the hematocrit of blood in large vessels ($\cong$0.45) for estimation of the input CA concentration in blood plasma [10], and $V_{T}$, $F/V_{T}$, ${F_{A}}/{V_{T}}$,${F_{\mathrm{PV}}}/{V_{T}}$, $v_{P}$, $v_{I}$, $t_{Lag,T}$, $R_{T}\left( t \right)$, $Q_{T}\left( t \right)$, $Q_{P}\left( t \right)$, $Q_{I}\left( t \right)$, $\bar{C}_{P}\left( t \right)$ and $\bar{C}_{I}\left( t \right)$ are kinetic parameters and functions as defined in Table 1. The fundamental assumption behind Equation (3) is that the CA transport within the capillary-tissue system can be modeled as a linear and time-invariant (stationary) system. All models considered in this study fall under this assumption. All five models are derived from their own tissue residue function $R_{T}\left( t \right)$. To account for the difference in bolus arrival times between the feeding vessels (i.e., hepatic artery and portal vein) and the liver tissue, a time lag (delay) to the liver tissue, $t_{Lag,T}$, can be imposed on either the net input function (i.e., $C_{A}\left( t \right)$ and $C_{\mathrm{PV}}\left( t \right)$) or $R_{T}\left( t \right)$ for calculation of $C_{T}\left( t \right)$. The analytic solution of $C_{T}\left( t \right)$ for each model can be derived by incorporation of Equations (1) and (2) into Equation (3).

The 2CX model is a conventional compartment model [8,10,12], which assumes instantaneously well-mixed (homogeneous) compartments, i.e., the movement of CA is sufficiently fast and distributes evenly throughout the compartment, so that the CA concentration is a function only of time $t$, but not of space. Thus, the 2CX model is a type of lumped-parameter model. A pair of differential equations for the two interacting compartments can be given by

$$\frac{d\bar{C}_{P}\left( t \right)}{dt}=\frac{F}{V_{P}}\left[ \frac{\gamma C_{A}\left( t-t_{Lag,T} \right)+\left( 1-\gamma\right)C_{PV}\left( t-t_{Lag,T} \right)}{1-H_{LV}}-C_{P}\left( t \right) \right]-\frac{PS}{V_{P}}\left[ \bar{C}_{P}\left( t \right)-\bar{C}_{I}\left( t \right) \right],$$

(4)


and

$$\frac{d\bar{C}_{I}\left( t \right)}{dt}=\frac{PS}{V_{I}}\left[ \bar{C}_{P}\left( t \right)-\bar{C}_{I}\left( t \right) \right],$$

(5)


where $PS$, $V_{P}$, and $V_{I}$ are defined in Table 1. The total tissue concentration of the 2CX model is given by $C_{T}\left( t \right)=v_{P}\bar{C}_{P}\left( t \right)+v_{I}\bar{C}_{I}\left( t \right)$, where $v_{P}={V_{P}}/{V_{T}}$ and $v_{I}={V_{I}}/{V_{T}}$. Thus, the solution of the tissue residue function of the 2CX model, $R_{T,2CX}\left( t \right)$, is given by

$$R_{T,2CX}\left( t \right)=Ae^{\alpha t}+\left( 1-A \right)e^{\beta t},$$

(6)


which is a bi-exponential function with

$$\left( \begin{aligned} \alpha\\ \beta\end{aligned} \right)=\frac{1}{2}\left[ -\left\{ \frac{F}{V_{P}}+\left( 1+\frac{v_{P}}{v_{I}} \right)\frac{PS}{V_{P}} \right\}\pm\sqrt{\left\{ \frac{F}{V_{P}}+\left( 1+\frac{v_{P}}{v_{I}} \right)\frac{PS}{V_{P}} \right\}^{2}-4\frac{v_{P}}{v_{I}}\frac{F}{V_{P}}\frac{PS}{V_{P}}} \right],$$

(7)


and

$$A=\frac{\alpha+\left( 1+\frac{v_{P}}{v_{I}} \right)\frac{PS}{V_{P}}}{\alpha-\beta}.$$

(8)


The impulse-response functions of the plasma and interstitial compartments of the 2CX model, $Q_{P,2CX}\left( t \right)$ and $Q_{I,2CX}\left( t \right)$, are

$$Q_{P,2CX}\left( t \right)=\frac{F}{V_{P}}\left\{ Be^{\alpha t}+\left( 1-B \right)e^{\beta t} \right\},$$

(9)


and

$$Q_{I,2CX}\left( t \right)=\frac{v_{P}}{v_{I}}\frac{F}{V_{P}}\left( A-B \right)\left( e^{\alpha t}-e^{\beta t} \right),$$

(10)


with

$$B=A-\frac{\frac{PS}{V_{P}}}{\alpha-\beta}.$$

(11)


If the functional expressions for $C_{A}\left( t \right)$ and $C_{\mathrm{PV}}\left( t \right)$ are assumed to take the form of Equations (1) and (2), respectively, the spatially averaged plasma and interstitial concentrations of the 2CX model, $\bar{C}_{P,2CX}\left( t \right)$ and $\bar{C}_{I,2CX}\left( t \right)$, are given by

$$\bar{C}_{P,2CX}\left( t \right)=\frac{F}{V_{P}}\left( \frac{1}{1-H_{LV}} \right)\left[ \gamma\left\{ \left( {K_{A,P1}e}^{\alpha t_{A1}}+{K_{A,P2}e}^{\beta t_{A1}}+K_{A,P3}e^{{-\mu}_{B,A}t_{A1}}+K_{A,P4}t_{A1}e^{{-\mu}_{B,A}t_{A1}} \right)u\left( t_{A1} \right)+\left( K_{A,P5}e^{\alpha t_{A2}}+K_{A,P6}e^{\beta t_{A2}}+K_{A,P7}e^{{-\mu}_{B,A}t_{A2}}+{K_{A,P8}e}^{{-\mu}_{G,A}t_{A2}}+K_{A,P9}t_{A2}e^{{-\mu}_{B,A}t_{A2}} \right)u\left( t_{A2} \right) \right\}+\left( 1-\gamma\right)\left\{ \left( {K_{PV,P1}e}^{\alpha t_{PV1}}+{K_{PV,P2}e}^{\beta t_{PV1}}+K_{PV,P3}e^{{-\mu}_{B,PV}t_{PV1}}+K_{PV,P4}t_{PV1}e^{{-\mu}_{B,PV}t_{PV1}} \right)u\left( t_{PV1} \right)+\left( K_{PV,P5}e^{\alpha t_{PV2}}+K_{PV,P6}e^{\beta t_{PV2}}+K_{PV,P7}e^{{-\mu}_{B,PV}t_{PV2}}+{K_{PV,P8}e}^{{-\mu}_{G,PV}t_{PV2}}+K_{PV,P9}t_{PV2}e^{{-\mu}_{B,PV}t_{PV2}} \right)u\left( t_{PV2} \right) \right\} \right],$$

(12)


and

$$\bar{C}_{I,2CX}\left( t \right)=\frac{v_{P}}{v_{I}}\frac{F}{V_{P}}\left( \frac{1}{1-H_{LV}} \right)\left[ \gamma\left\{ \left( {K_{A,I1}e}^{\alpha t_{A1}}+{K_{A,I2}e}^{\beta t_{A1}}+K_{A,I3}e^{{-\mu}_{B,A}t_{A1}}+K_{A,I4}t_{A1}e^{{-\mu}_{B,A}t_{A1}} \right)u\left( t_{A1} \right)+\left( K_{A,I5}e^{\alpha t_{A2}}+K_{A,I6}e^{\beta t_{A2}}+K_{A,I7}e^{{-\mu}_{B,A}t_{A2}}+{K_{A,I8}e}^{{-\mu}_{G,A}t_{A2}}+K_{A,I9}t_{A2}e^{{-\mu}_{B,A}t_{A2}} \right)u\left( t_{A2} \right) \right\}+\left( 1-\gamma\right)\left\{ \left( {K_{PV,I1}e}^{\alpha t_{PV1}}+{K_{PV,I2}e}^{\beta t_{PV1}}+K_{PV,I3}e^{{-\mu}_{B,PV}t_{PV1}}+K_{PV,I4}t_{PV1}e^{{-\mu}_{B,PV}t_{PV1}} \right)u\left( t_{PV1} \right)+\left( K_{PV,I5}e^{\alpha t_{PV2}}+K_{PV,I6}e^{\beta t_{PV2}}+K_{PV,I7}e^{{-\mu}_{B,PV}t_{PV2}}+{K_{PV,I8}e}^{{-\mu}_{G,PV}t_{PV2}}+K_{PV,I9}t_{PV2}e^{{-\mu}_{B,PV}t_{PV2}} \right)u\left( t_{PV2} \right) \right\} \right],$$

(13)


where $t_{A1}=t-t_{Lag,A1}-t_{Lag,T}$, $t_{A2}=t_{A1}-t_{Lag,A2}$, $t_{PV1}=t-t_{Lag,PV1}-t_{Lag,T}$, $t_{PV2}=t_{PV1}-t_{Lag,PV2}$, $K_{A,P1}=B{a_{B,A}}/{\left( \alpha+\mu_{B,A} \right)^{2}}$, $K_{A,P2}=\left( 1-B \right){a_{B,A}}/{\left( \beta+\mu_{B,A} \right)^{2}}$, $K_{A,P3}=-\left( K_{A,P1}+K_{A,P2} \right)$, $K_{A,P4}=-\left\{ K_{A,P1}\left( \alpha+\mu_{B,A} \right)+K_{A,P2}\left( \beta+\mu_{B,A} \right) \right\}$, $K_{A,P5}=K_{A,P1}{a_{G,A}}/\left( \alpha+\mu_{G,A} \right)$, $K_{A,P6}=K_{A,P2}{a_{G,A}}/\left( \beta+\mu_{G,A} \right)$, $K_{A,P7}=\left\{ {a_{G,A}}/{\left( \mu_{B,A}-\mu_{G,A} \right)^{2}} \right\}\left\{ K_{A,P1}\left( \alpha+2\mu_{B,A}-\mu_{G,A} \right)+K_{A,P2}\left( \beta+2\mu_{B,A}-\mu_{G,A} \right) \right\}$, $K_{A,P8}=-\left\{ {a_{B,A}}/{\left( \mu_{B,A}-\mu_{G,A} \right)^{2}} \right\}\left\{ B\left( {K_{A,P5}}/{K_{A,P1}} \right)+\left( 1-B \right)\left( {K_{A,P6}}/{K_{A,P2}} \right) \right\}$, $K_{A,P9}=-K_{A,P4}{a_{G,A}}/\left( \mu_{B,A}-\mu_{G,A} \right)$, $K_{PV,P1}=B{a_{B,PV}}/{\left( \alpha+\mu_{B,PV} \right)^{2}}$, $K_{PV,P2}=\left( 1-B \right){a_{B,PV}}/{\left( \beta+\mu_{B,PV} \right)^{2}}$, $K_{PV,P3}=-\left( K_{PV,P1}+K_{PV,P2} \right)$, $K_{PV,P4}=-\left\{ K_{PV,P1}\left( \alpha+\mu_{B,PV} \right)+K_{PV,P2}\left( \beta+\mu_{B,PV} \right) \right\}$, $K_{PV,P5}=K_{PV,P1}{a_{G,PV}}/\left( \alpha+\mu_{G,PV} \right)$, $K_{PV,P6}=K_{PV,P2}{a_{G,PV}}/\left( \beta+\mu_{G,PV} \right)$, $K_{PV,P7}=\left\{ {a_{G,PV}}/{\left( \mu_{B,PV}-\mu_{G,PV} \right)^{2}} \right\}\left\{ K_{PV,P1}\left( \alpha+2\mu_{B,PV}-\mu_{G,PV} \right)+K_{PV,P2}\left( \beta+2\mu_{B,PV}-\mu_{G,PV} \right) \right\}$, $K_{PV,P8}=-\left\{ {a_{B,PV}}/{\left( \mu_{B,PV}-\mu_{G,PV} \right)^{2}} \right\}\left\{ B\left( {K_{PV,P5}}/{K_{PV,P1}} \right)+\left( 1-B \right)\left( {K_{PV,P6}}/{K_{PV,P2}} \right) \right\}$, $K_{PV,P9}=-K_{PV,P4}{a_{G,PV}}/\left( \mu_{B,PV}-\mu_{G,PV} \right)$, $K_{A,I1}=\left( A-B \right){a_{B,A}}/{\left( \alpha+\mu_{B,A} \right)^{2}}$, $K_{A,I2}=\left( A-B \right){a_{B,A}}/{\left( \beta+\mu_{B,A} \right)^{2}}$, $K_{A,I3}=-\left( K_{A,I1}+K_{A,I2} \right)$, $K_{A,I4}=-\left\{ K_{A,I1}\left( \alpha+\mu_{B,A} \right)+K_{A,I2}\left( \beta+\mu_{B,A} \right) \right\}$, $K_{A,I5}=K_{A,I1}{a_{G,A}}/\left( \alpha+\mu_{G,A} \right)$, $K_{A,I6}=K_{A,I2}{a_{G,A}}/\left( \beta+\mu_{G,A} \right)$, $K_{A,I7}=\left\{ {a_{G,A}}/{\left( \mu_{B,A}-\mu_{G,A} \right)^{2}} \right\}\left\{ K_{A,I1}\left( \alpha+2\mu_{B,A}-\mu_{G,A} \right)+K_{A,I2}\left( \beta+2\mu_{B,A}-\mu_{G,A} \right) \right\}$, $K_{A,I8}=-\left( A-B \right)\left\{ {a_{B,A}}/{\left( \mu_{B,A}-\mu_{G,A} \right)^{2}} \right\}\left( {K_{A,I5}}/{K_{A,I1}}+{K_{A,I6}}/{K_{A,I2}} \right)$, $K_{A,I9}=-K_{A,I4}{a_{G,A}}/\left( \mu_{B,A}-\mu_{G,A} \right)$, $K_{PV,I1}=\left( A-B \right){a_{B,PV}}/{\left( \alpha+\mu_{B,PV} \right)^{2}}$, $K_{PV,I2}=\left( A-B \right){a_{B,PV}}/{\left( \beta+\mu_{B,PV} \right)^{2}}$, $K_{PV,I3}=-\left( K_{PV,I1}+K_{PV,I2} \right)$, $K_{PV,I4}=-\left\{ K_{PV,I1}\left( \alpha+\mu_{B,PV} \right)+K_{PV,I2}\left( \beta+\mu_{B,PV} \right) \right\}$, $K_{PV,I5}=K_{PV,I1}{a_{G,PV}}/\left( \alpha+\mu_{G,PV} \right)$, $K_{PV,I6}=K_{PV,I2}{a_{G,PV}}/\left( \beta+\mu_{G,PV} \right)$, $K_{PV,I7}=\left\{ {a_{G,PV}}/{\left( \mu_{B,PV}-\mu_{G,PV} \right)^{2}} \right\}\left\{ K_{PV,I1}\left( \alpha+2\mu_{B,PV}-\mu_{G,PV} \right)+K_{PV,I2}\left( \beta+2\mu_{B,PV}-\mu_{G,PV} \right) \right\}$, $K_{PV,I8}=-\left( A-B \right)\left\{ {a_{B,PV}}/{\left( \mu_{B,PV}-\mu_{G,PV} \right)^{2}} \right\}\left( {K_{PV,I5}}/{K_{PV,I1}}+{K_{PV,I6}}/{K_{PV,I2}} \right)$, and $K_{PV,I9}=-K_{PV,I4}{a_{G,PV}}/\left( \mu_{B,PV}-\mu_{G,PV} \right)$.

The TH model describes the capillary bed represented by a single cylinder of length $L$, which contains the plasma volume $V_{P}$ [8,12,13]. The interstitial space is assumed to be a cylindrical annulus around the capillary, with the interstitial volume $V_{I}$. The plasma concentration of CA, $C_{P}\left( x,t \right)$, is a function of both the axial position $x$ along the capillary and the time $t$. The concentration of the interstitial space, $C_{I}\left( t \right)$, depends only on time; in other words, the interstitial space is treated as a well-mixed compartment. Thus, the mass conservation for the interstitial space is the same as that of the 2CX model (Equation (5)). The differential equations that describe the TH model are expressed as follows:

$$\frac{\partial C_{P}\left( x,t \right)}{\partial t}=\frac{F}{V_{P}}\left[ \frac{\left\{ \gamma C_{A}\left( t-t_{Lag,T} \right)+\left( 1-\gamma\right)C_{PV}\left( t-t_{Lag,T} \right) \right\}\delta\left( x \right)}{1-H_{LV}}-L\frac{\partial C_{P}\left( x,t \right)}{\partial x} \right]-\frac{PS}{V_{P}}\left[ C_{P}\left( x,t \right)-C_{I}\left( t \right) \right],$$

(14)


and

$$\frac{dC_{I}\left( t \right)}{dt}=\frac{1}{L}\frac{PS}{V_{I}}\int_{0}^{L} \left[ C_{P}\left( x,t \right)-C_{I}\left( t \right) \right]dx,$$

(15)


where $\delta\left( x \right)$ is the Dirac delta function that denotes the idealized impulse excitation of a unit-mass source at $x=0$. It should be noted that there exists no known closed-form solution for the system of Equations (14) and (15) in the time domain.

The AATH model describes a closed-form solution of the TH model in the time domain with use of an adiabatic approximation [8,12,14]. In contrast to the TH model, the capillary walls are assumed to be impermeable to CA in the AATH model, and the interstitial space receives influx with clearance $EF$ from the venous end of the capillary. The remaining fraction, $\left( 1-E \right)F$, and the outflux from the interstitial space drain into the vein. Because the capillary walls are impermeable to CA in the AATH model, the equation of CA mass conservation in the capillary bed is the plug flow equation without decay, which accounts for spatial differences in the plasma concentration. Because $C_{P}\left( L,t \right)$ is the concentration at the venous end of the capillary, the equation for the interstitial space is that of a compartment with influx $EFC_{P}\left( L,t \right)$. This leads to the following pair of equations:

$$\frac{\partial C_{P}\left( x,t \right)}{\partial t}=\frac{F}{V_{P}}\left[ \frac{\left\{ \gamma C_{A}\left( t-t_{Lag,T} \right)+\left( 1-\gamma\right)C_{PV}\left( t-t_{Lag,T} \right) \right\}\delta\left( x \right)}{1-H_{LV}}-L\frac{\partial C_{P}\left( x,t \right)}{\partial x} \right]$$

(16)


and

$$\frac{dC_{I}\left( t \right)}{dt}=\frac{EF}{V_{I}}\left[ C_{P}\left( L,t \right)-C_{I}\left( t \right) \right].$$

(17)


The total tissue concentration of the AATH model is given by $C_{T}\left( t \right)=v_{P}\int_{0}^{L} C_{P}\left( x,t \right)dx+v_{I}LC_{I}\left( t \right)$, where $\bar{C}_{P}\left( t \right)=\int_{0}^{L} C_{P}\left( x,t \right)dx$ and $\bar{C}_{I}\left( t \right)=LC_{I}\left( t \right)$. The corresponding tissue residue function for the AATH model, $R_{T,AATH}\left( t \right)$, is given by

$$R_{T,AATH}\left( t \right)=u\left( t \right)+\left[ Ee^{-\frac{v_{P}}{v_{I}}\frac{EF}{V_{P}}\left( t-\frac{V_{P}}{F} \right)}-1 \right]u\left( t-\frac{V_{P}}{F} \right).$$

(18)


The impulse response functions of the plasma and interstitial compartments of the AATH model, $Q_{P,AATH}\left( t \right)$ and $Q_{I,AATH}\left( t \right)$, are

$$Q_{P,AATH}\left( t \right)=\frac{F}{V_{P}}\left\{ u\left( t \right)-u\left( t-\frac{V_{P}}{F} \right) \right\}$$

(19)


and

$$Q_{I,AATH}\left( t \right)=\frac{v_{P}}{v_{I}}\frac{EF}{V_{P}}u\left( t-\frac{V_{P}}{F} \right)e^{-\frac{v_{P}}{v_{I}}\frac{EF}{V_{P}}\left( t-\frac{V_{P}}{F} \right)}.$$

(20)


The spatially averaged plasma and interstitial concentrations of the AATH model, $\bar{C}_{P,AATH}\left( t \right)$ and $\bar{C}_{I,AATH}\left( t \right)$, are given by

$$\bar{C}_{P,AATH}\left( t \right)=\frac{F}{V_{P}}\left( \frac{1}{1-H_{LV}} \right)\left[ \gamma\left[ \left\{ L_{A,P1}\left( 1-e^{{-\mu}_{B,A}t_{A1}} \right)+L_{A,P2}t_{A1}e^{{-\mu}_{B,A}t_{A1}} \right\}u\left( t_{A1} \right)+\left( L_{A,P3}+L_{A,P4}e^{{-\mu}_{B,A}t_{A2}}+{L_{A,P5}e}^{{-\mu}_{G,A}t_{A2}}+L_{A,P6}t_{A2}e^{{-\mu}_{B,A}t_{A2}} \right)u\left( t_{A2} \right)-\left\{ L_{A,P1}\left( 1-e^{{-\mu}_{B,A}\left( t_{A1}-\frac{V_{P}}{F} \right)} \right)+L_{A,P2}\left( t_{A1}-\frac{V_{P}}{F} \right)e^{{-\mu}_{B,A}\left( t_{A1}-\frac{V_{P}}{F} \right)} \right\}u\left( t_{A1}-\frac{V_{P}}{F} \right)-\left\{ L_{A,P3}+L_{A,P4}e^{{-\mu}_{B,A}\left( t_{A2}-\frac{V_{P}}{F} \right)}+{L_{A,P5}e}^{{-\mu}_{G,A}\left( t_{A2}-\frac{V_{P}}{F} \right)}+L_{A,P6}\left( t_{A2}-\frac{V_{P}}{F} \right)e^{{-\mu}_{B,A}\left( t_{A2}-\frac{V_{P}}{F} \right)} \right\}u\left( t_{A2}-\frac{V_{P}}{F} \right) \right]+\left( 1-\gamma\right)\left[ \left\{ L_{PV,P1}\left( 1-e^{{-\mu}_{B,PV}t_{PV1}} \right)+L_{PV,P2}t_{PV1}e^{{-\mu}_{B,PV}t_{PV1}} \right\}u\left( t_{PV1} \right)+\left( L_{PV,P3}+L_{PV,P4}e^{{-\mu}_{B,PV}t_{PV2}}+{L_{PV,P5}e}^{{-\mu}_{G,PV}t_{PV2}}+L_{PV,P6}t_{PV2}e^{{-\mu}_{B,PV}t_{PV2}} \right)u\left( t_{PV2} \right)-\left\{ L_{PV,P1}\left( 1-e^{{-\mu}_{B,PV}\left( t_{PV1}-\frac{V_{P}}{F} \right)} \right)+L_{PV,P2}\left( t_{PV1}-\frac{V_{P}}{F} \right)e^{{-\mu}_{B,PV}\left( t_{PV1}-\frac{V_{P}}{F} \right)} \right\}u\left( t_{PV1}-\frac{V_{P}}{F} \right)-\left\{ L_{PV,P3}+L_{PV,P4}e^{{-\mu}_{B,PV}\left( t_{PV2}-\frac{V_{P}}{F} \right)}+{L_{PV,P5}e}^{{-\mu}_{G,PV}\left( t_{PV2}-\frac{V_{P}}{F} \right)}+L_{PV,P6}\left( t_{PV2}-\frac{V_{P}}{F} \right)e^{{-\mu}_{B,PV}\left( t_{PV2}-\frac{V_{P}}{F} \right)} \right\}u\left( t_{PV2}-\frac{V_{P}}{F} \right) \right] \right],$$

(21)


and

$$\bar{C}_{I,AATH}\left( t \right)=\frac{v_{P}}{v_{I}}\frac{F}{V_{P}}\left( \frac{1}{1-H_{LV}} \right)\left[ \gamma\left[ \left\{ L_{A,I1}\left( e^{-\frac{v_{P}}{v_{I}}\frac{EF}{V_{P}}\left( t_{A1}-\frac{V_{P}}{F} \right)}-e^{{-\mu}_{B,A}\left( t_{A1}-\frac{V_{P}}{F} \right)} \right)+L_{A,I2}\left( t_{A1}-\frac{V_{P}}{F} \right)e^{{-\mu}_{B,A}\left( t_{A1}-\frac{V_{P}}{F} \right)} \right\}u\left( t_{A1}-\frac{V_{P}}{F} \right)+\left\{ L_{A,I3}e^{-\frac{v_{P}}{v_{I}}\frac{EF}{V_{P}}\left( t_{A2}-\frac{V_{P}}{F} \right)}+L_{A,I4}e^{{-\mu}_{B,A}\left( t_{A2}-\frac{V_{P}}{F} \right)}+{L_{A,I5}e}^{{-\mu}_{G,A}\left( t_{A2}-\frac{V_{P}}{F} \right)}+L_{A,I6}\left( t_{A2}-\frac{V_{P}}{F} \right)e^{{-\mu}_{B,A}\left( t_{A2}-\frac{V_{P}}{F} \right)} \right\}u\left( t_{A2}-\frac{V_{P}}{F} \right) \right]+\left( 1-\gamma\right)\left[ \left\{ L_{PV,I1}\left( e^{-\frac{v_{P}}{v_{I}}\frac{EF}{V_{P}}\left( t_{PV1}-\frac{V_{P}}{F} \right)}-e^{{-\mu}_{B,PV}\left( t_{PV1}-\frac{V_{P}}{F} \right)} \right)+L_{PV,I2}\left( t_{PV1}-\frac{V_{P}}{F} \right)e^{{-\mu}_{B,PV}\left( t_{PV1}-\frac{V_{P}}{F} \right)} \right\}u\left( t_{PV1}-\frac{V_{P}}{F} \right)+\left\{ L_{PV,I3}e^{-\frac{v_{P}}{v_{I}}\frac{EF}{V_{P}}\left( t_{PV2}-\frac{V_{P}}{F} \right)}+L_{PV,I4}e^{{-\mu}_{B,PV}\left( t_{PV2}-\frac{V_{P}}{F} \right)}+{L_{PV,I5}e}^{{-\mu}_{G,PV}\left( t_{PV2}-\frac{V_{P}}{F} \right)}+L_{PV,I6}\left( t_{PV2}-\frac{V_{P}}{F} \right)e^{{-\mu}_{B,PV}\left( t_{PV2}-\frac{V_{P}}{F} \right)} \right\}u\left( t_{PV2}-\frac{V_{P}}{F} \right) \right] \right],$$

(22)


where $L_{A,P1}={a_{B,A}}/{{\mu_{B,A}}^{2}}$, $L_{A,P2}=-L_{A,P1}\mu_{B,A}$, $L_{A,P3}=L_{A,P1}{a_{G,A}}/{\mu_{G,A}}$, $L_{A,P4}=\left\{ {a_{G,A}}/\left( \mu_{B,A}-\mu_{G,A} \right) \right\}\left\{ L_{A,P1}-{L_{A,P2}}/\left( \mu_{B,A}-\mu_{G,A} \right) \right\}$, $L_{A,P5}=-{a_{B,A}a_{G,A}}/\left\{ \mu_{G,A}\left( \mu_{B,A}-\mu_{G,A} \right)^{2} \right\}$, $L_{A,P6}=-L_{A,P2}{a_{G,A}}/\left( \mu_{B,A}-\mu_{G,A} \right)$, $L_{PV,P1}={a_{B,PV}}/{{\mu_{B,PV}}^{2}}$, $L_{PV,P2}=-L_{PV,P1}\mu_{B,PV}$, $L_{PV,P3}=L_{PV,P1}{a_{G,PV}}/{\mu_{G,PV}}$, $L_{PV,P4}=\left\{ {a_{G,PV}}/\left( \mu_{B,PV}-\mu_{G,PV} \right) \right\}\left\{ L_{PV,P1}-{L_{PV,P2}}/\left( \mu_{B,PV}-\mu_{G,PV} \right) \right\}$, $L_{PV,P5}=-{a_{B,PV}a_{G,PV}}/\left\{ \mu_{G,PV}\left( \mu_{B,PV}-\mu_{G,PV} \right)^{2} \right\}$, $L_{PV,P6}=-L_{PV,P2}{a_{G,PV}}/\left( \mu_{B,PV}-\mu_{G,PV} \right)$, $L_{A,I1}={Ea_{B,A}}/{\left( \mu_{B,A}-L_{1} \right)^{2}}$, $L_{A,I2}=-L_{A,I1}\left( \mu_{B,A}-L_{1} \right)$, $L_{A,I3}=L_{A,I1}{a_{G,A}}/\left( \mu_{G,A}-L_{1} \right)$, $L_{A,I4}=L_{A,I1}\left\{ {a_{G,A}}/\left( \mu_{B,A}-\mu_{G,A} \right) \right\}\left\{ 1+\left( \mu_{B,A}-L_{1} \right)/\left( \mu_{B,A}-\mu_{G,A} \right) \right\}$, $L_{A,I5}=-L_{A,I3}\left\{ \left( \mu_{B,A}-L_{1} \right)/\left( \mu_{B,A}-\mu_{G,A} \right) \right\}^{2}$, $L_{A,I6}=-L_{A,I2}{a_{G,A}}/\left( \mu_{B,A}-\mu_{G,A} \right)$, $L_{PV,I1}={Ea_{B,PV}}/{\left( \mu_{B,PV}-L_{1} \right)^{2}}$, $L_{PV,I2}=-L_{PV,I1}\left( \mu_{B,PV}-L_{1} \right)$, $L_{PV,I3}=L_{PV,I1}{a_{G,PV}}/\left( \mu_{G,PV}-L_{1} \right)$, $L_{PV,I4}=L_{PV,I1}\left\{ {a_{G,PV}}/\left( \mu_{B,PV}-\mu_{G,PV} \right) \right\}\left\{ 1+\left( \mu_{B,PV}-L_{1} \right)/\left( \mu_{B,PV}-\mu_{G,PV} \right) \right\}$, and $L_{PV,I5}=-L_{PV,I3}\left\{ \left( \mu_{B,PV}-L_{1} \right)/\left( \mu_{B,PV}-\mu_{G,PV} \right) \right\}^{2}$, $L_{PV,I6}=-L_{PV,I2}{a_{G,PV}}/\left( \mu_{B,PV}-\mu_{G,PV} \right)$, where $L_{1}=\left( {v_{P}}/{v_{I}} \right)/\left( {EF}/{V_{P}} \right)$.

The DP model describes the capillary bed as a plug-flow system like the AATH model [8,12,15]. However, unlike the AATH model, the interstitial compartment is modeled as a series of infinitesimal compartments that exchange CA only with nearby locations in the capillary bed. Thus, the CA concentrations $C_{P}\left( x,t \right)$ and $C_{I}\left( x,t \right)$ both rely on the position of a capillary tube. The DP model does not allow for axial CA transport in the interstitial space, and thereby the CA cannot travel to the venous ends of the capillary bed through the interstitial space. Therefore, the DP model can be considered as a chain of infinitesimal 2CX models. The mass-balance equations for the DP model can be formulated for an elemental volume $dx$ along the axial length $L$ of a capillary tube as follows:

$$\frac{\partial C_{P}\left( x,t \right)}{\partial t}=\frac{F}{V_{P}}\left[ \frac{\left\{ \gamma C_{A}\left( t-t_{Lag,T} \right)+\left( 1-\gamma\right)C_{PV}\left( t-t_{Lag,T} \right) \right\}\delta\left( x \right)}{1-H_{LV}}-L\frac{\partial C_{P}\left( x,t \right)}{\partial x} \right]-\frac{PS}{V_{P}}\left[ C_{P}\left( x,t \right)-C_{I}\left( x,t \right) \right]$$

(23)


and

$$\frac{\partial C_{I}\left( x,t \right)}{\partial t}=\frac{PS}{V_{I}}\left[ C_{P}\left( x,t \right)-C_{I}\left( x,t \right) \right].$$

(24)


The total tissue concentration of the DP model is given by $C_{T}\left( t \right)=v_{P}\int_{0}^{L} C_{P}\left( x,t \right)dx+v_{I}\int_{0}^{L} C_{I}\left( x,t \right)dx$, where $\bar{C}_{P}\left( t \right)=\int_{0}^{L} C_{P}\left( x,t \right)dx$ and $\bar{C}_{I}\left( t \right)=\int_{0}^{L} C_{I}\left( x,t \right)dx$. The corresponding tissue residue function for the DP model, $R_{T,DP}\left( t \right)$, is given by

$$R_{T,DP}\left( t \right)=u\left( t \right)-e^{-\frac{PS}{F}}\left[ 1+\frac{PS}{V_{P}}\int_{0}^{t-\frac{V_{P}}{F}} e^{-\frac{v_{P}}{v_{I}}\frac{PS}{V_{P}}\tau}\sqrt{\frac{v_{P}}{v_{I}}\frac{V_{P}}{F}\frac{1}{\tau}}I_{1}\left( 2\frac{PS}{V_{P}}\sqrt{\frac{v_{P}}{v_{I}}\frac{V_{P}}{F}\tau} \right)d\tau\right]u\left( t-\frac{V_{P}}{F} \right),$$

(25)


where $I_{1}\left( t \right)$ denotes a modified Bessel function of the first kind. The integral term including the modified Bessel function cannot be solved into a fully analytic form. Although the integral term can be calculated by numerical integration (e.g., with the Kutta-Merson method), it is computationally expensive. To simplify the formulation of the integral term in Equation (25), an alternative derivation can be considered by evaluation of a Taylor series expansion [16]. The first two terms of the Taylor series may be sufficient for extracting identifiable parameters in the parenchyma phase because they contain information on both ${PS}/{V_{P}}$ and ${PS}/{V_{I}}$. The $R_{T,DP}\left( t \right)$ can be simplified as

$$R_{T,DP}\left( t \right)\cong u\left( t \right)-e^{-\frac{PS}{F}}\left[ 1+\frac{v_{P}}{v_{I}}\frac{PS}{V_{P}}\frac{PS}{F}\left( t-\frac{V_{P}}{F} \right) \right]u\left( t-\frac{V_{P}}{F} \right).$$

(26)


The impulse response functions of the plasma and interstitial compartments of the DP model, $Q_{P,DP}\left( t \right)$ and $Q_{I,DP}\left( t \right)$, are

$$Q_{P,DP}\left( t \right)=Q_{P,AATH}\left( t \right)$$

(27)


and

$$Q_{I,DP}\left( t \right)\cong\frac{v_{P}}{v_{I}}\frac{F}{V_{P}}u\left( t-\frac{V_{P}}{F} \right)\left[ 1-e^{-\frac{PS}{F}}\left\{ 1+\frac{v_{P}}{v_{I}}\frac{PS}{V_{P}}\frac{PS}{F}\left( t-\frac{V_{P}}{F} \right) \right\} \right].$$

(28)


The spatially averaged plasma and interstitial concentrations of the DP model, $\bar{C}_{P,DP}\left( t \right)$ and $\bar{C}_{I,DP}\left( t \right)$, are given by

$$\bar{C}_{P,DP}\left( t \right)=\bar{C}_{P,AATH}\left( t \right)$$

(29)


and

$$\bar{C}_{I,DP}\left( t \right)=\frac{v_{P}}{v_{I}}\frac{F}{V_{P}}\left( \frac{1}{1-H_{LV}} \right)\left[ \gamma\left[ \left\{ M_{A,I1}\left( 1-e^{{-\mu}_{B,A}\left( t_{A1}-\frac{V_{P}}{F} \right)} \right)+M_{A,I2}\left( t_{A1}-\frac{V_{P}}{F} \right)+M_{A,I3}\left( t_{A1}-\frac{V_{P}}{F} \right)e^{{-\mu}_{B,A}\left( t_{A1}-\frac{V_{P}}{F} \right)} \right\}u\left( t_{A1}-\frac{V_{P}}{F} \right)+\left\{ M_{A,I4}+M_{A,I5}e^{{-\mu}_{B,A}\left( t_{A2}-\frac{V_{P}}{F} \right)}+{M_{A,I6}e}^{{-\mu}_{G,A}\left( t_{A2}-\frac{V_{P}}{F} \right)}+M_{A,I7}\left( t_{A2}-\frac{V_{P}}{F} \right)+M_{A,I8}\left( t_{A2}-\frac{V_{P}}{F} \right)e^{{-\mu}_{B,A}\left( t_{A2}-\frac{V_{P}}{F} \right)} \right\}u\left( t_{A2}-\frac{V_{P}}{F} \right) \right]+\left( 1-\gamma\right)\left[ \left\{ M_{PV,I1}\left( 1-e^{{-\mu}_{B,PV}\left( t_{PV1}-\frac{V_{P}}{F} \right)} \right)+M_{PV,I2}\left( t_{PV1}-\frac{V_{P}}{F} \right)+M_{PV,I3}\left( t_{PV1}-\frac{V_{P}}{F} \right)e^{{-\mu}_{B,PV}\left( t_{PV1}-\frac{V_{P}}{F} \right)} \right\}u\left( t_{PV1}-\frac{V_{P}}{F} \right)+\left\{ M_{PV,I4}+M_{PV,I5}e^{{-\mu}_{B,PV}\left( t_{PV2}-\frac{V_{P}}{F} \right)}+{M_{PV,I6}e}^{{-\mu}_{G,PV}\left( t_{PV2}-\frac{V_{P}}{F} \right)}+M_{PV,I7}\left( t_{PV2}-\frac{V_{P}}{F} \right)+M_{PV,I8}\left( t_{PV2}-\frac{V_{P}}{F} \right)e^{{-\mu}_{B,PV}\left( t_{PV2}-\frac{V_{P}}{F} \right)} \right\}u\left( t_{PV2}-\frac{V_{P}}{F} \right) \right] \right],$$

(30)


where $M_{A,I1}=\left( {a_{B,A}}/{{\mu_{B,A}}^{2}} \right)\left( E+{2M_{1}}/{\mu_{B,A}} \right)$, $M_{A,I2}=-M_{1}{a_{B,A}}/{{\mu_{B,A}}^{2}}$, $M_{A,I3}=-\left( {a_{B,A}}/{\mu_{B,A}} \right)\left( E+{M_{1}}/{\mu_{B,A}} \right)$, $M_{A,I4}=\left( {a_{G,A}}/{\mu_{G,A}} \right)\left( M_{A,I1}-{M_{A,I2}}/{\mu_{G,A}} \right)$, $M_{A,I5}=-\left\{ \left( {a_{G,A}}/{\mu_{B,A}} \right)/\left( \mu_{B,A}-\mu_{G,A} \right) \right\}\left[ M_{A,I3}\left\{ 1+{\mu_{B,A}}/\left( \mu_{B,A}-\mu_{G,A} \right) \right\}+M_{A,I2} \right]$, $M_{A,I6}=-\left( E+{M_{1}}/{\mu_{G,A}} \right)\left( {a_{B,A}a_{G,A}}/{\mu_{G,A}} \right)/{\left( \mu_{B,A}-\mu_{G,A} \right)^{2}}$, $M_{A,I7}=M_{A,I2}{a_{G,A}}/{\mu_{G,A}}$, $M_{A,I8}=-M_{A,I3}{a_{G,A}}/\left( \mu_{B,A}-\mu_{G,A} \right)$, $M_{PV,I1}=\left( {a_{B,PV}}/{{\mu_{B,PV}}^{2}} \right)\left( E+{2M_{1}}/{\mu_{B,PV}} \right)$, $M_{PV,I2}=-M_{1}{a_{B,PV}}/{{\mu_{B,PV}}^{2}}$, $M_{PV,I3}=-\left( {a_{B,PV}}/{\mu_{B,PV}} \right)\left( E+{M_{1}}/{\mu_{B,PV}} \right)$, $M_{PV,I4}=\left( {a_{G,PV}}/{\mu_{G,PV}} \right)\left( M_{PV,I1}-{M_{PV,I2}}/{\mu_{G,PV}} \right)$, $M_{PV,I5}=-\left\{ \left( {a_{G,PV}}/{\mu_{B,PV}} \right)/\left( \mu_{B,PV}-\mu_{G,PV} \right) \right\}\left[ M_{PV,I3}\left\{ 1+{\mu_{B,PV}}/\left( \mu_{B,PV}-\mu_{G,PV} \right) \right\}+M_{PV,I2} \right]$, $M_{PV,I6}=-\left( E+{M_{1}}/{\mu_{G,PV}} \right)\left( {a_{B,PV}a_{G,PV}}/{\mu_{G,PV}} \right)/{\left( \mu_{B,PV}-\mu_{G,PV} \right)^{2}}$, $M_{PV,I7}=M_{PV,I2}{a_{G,PV}}/{\mu_{G,PV}}$, and $M_{PV,I8}=-M_{PV,I3}{a_{G,PV}}/\left( \mu_{B,PV}-\mu_{G,PV} \right)$, where $M_{1}=\left( {v_{P}}/{v_{I}} \right)\left( {PS}/{V_{P}} \right)\left( {PS}/F \right)e^{-\left( {PS}/F \right)}$.

The TK model considered in this study is a reduced version of the 2CX model with a mixed flow- and permeability-limited condition [17]. To simplify the second order system of Equations (4) and (5), the change in $\bar{C}_{P}\left( t \right)$ must be slow compared to the transit time of CA [18]. Taking ${d\bar{C}_{P}\left( t \right)}/{dt}\cong0$, Equations (4) and (5) become, respectively,

$$\bar{C}_{P}\left( t \right)=\frac{\left\{ \gamma C_{A}\left( t-t_{Lag,T} \right)+\left( 1-\gamma\right)C_{PV}\left( t-t_{Lag,T} \right) \right\}/\left( 1-H_{LV} \right)+{\left( {PS}/F \right)\bar{C}}_{I}\left( t \right)}{1+{PS}/F}$$

(31)


and

$$\frac{{d\bar{C}}_{I}\left( t \right)}{dt}=\frac{F}{V_{I}}\left( \frac{{PS}/F}{1+{PS}/F} \right)\left[ \frac{\gamma C_{A}\left( t-t_{Lag,T} \right)+\left( 1-\gamma\right)C_{PV}\left( t-t_{Lag,T} \right)}{1-H_{LV}}-\bar{C}_{I}\left( t \right) \right].$$

(32)


If $F\to\infty$ (permeability-limited regime), Equations (31) and (32) become, respectively,

$$\bar{C}_{P}\left( t \right)=\frac{\gamma C_{A}\left( t-t_{Lag,T} \right)+\left( 1-\gamma\right)C_{PV}\left( t-t_{Lag,T} \right)}{1-H_{LV}}$$

(33)


and

$$\frac{{d\bar{C}}_{I}\left( t \right)}{dt}=\frac{PS}{V_{I}}\left[ \frac{\gamma C_{A}\left( t-t_{Lag,T} \right)+\left( 1-\gamma\right)C_{PV}\left( t-t_{Lag,T} \right)}{1-H_{LV}}-\bar{C}_{I}\left( t \right) \right].$$

(34)


Conversely, if $PS\to\infty$ (flow-limited regime), Equations (31) and (32) become, respectively,

$$\bar{C}_{P}\left( t \right)=\bar{C}_{I}\left( t \right)$$

(35)


and

$$\frac{{d\bar{C}}_{I}\left( t \right)}{dt}=\frac{F}{V_{I}}\left[ \frac{\gamma C_{A}\left( t-t_{Lag,T} \right)+\left( 1-\gamma\right)C_{PV}\left( t-t_{Lag,T} \right)}{1-H_{LV}}-\bar{C}_{I}\left( t \right) \right].$$

(36)


The extraction fraction can be evaluated formally with the Rankin-Crone equation [19,20]: $E=1-e^{-\frac{PS}{F}},$ where $EF\cong PS$ in the permeability-limited regime, whereas $EF\cong F$ in the flow-limited regime. Consequently, the parameter $EF$ enables a compromise between the two regimes, i.e., the mixed flow- and permeability-limited condition. Assuming that $v_{P}\ll v_{I}$, and $C_{T}\left( t \right)\cong v_{I}\bar{C}_{I}\left( t \right)$, Equations (34) and (36) are converted into

$$\frac{{dC}_{T}\left( t \right)}{dt}=\frac{EF}{V_{T}}\left[ \frac{\gamma C_{A}\left( t-t_{Lag,T} \right)+\left( 1-\gamma\right)C_{PV}\left( t-t_{Lag,T} \right)}{1-H_{LV}}-\frac{C_{T}\left( t \right)}{v_{I}} \right].$$

(37)


The corresponding tissue residue function for the TK model, $R_{T,TK}\left( t \right)$, is

$$R_{T,TK}\left( t \right)=Ee^{-\frac{v_{P}}{v_{I}}\frac{EF}{V_{P}}t},$$

(38)


which is a single-exponential function. The impulse response function of the interstitial compartment of the TK model, $Q_{I,TK}\left( t \right)$, is

$$Q_{I,TK}\left( t \right)=\frac{v_{P}}{v_{I}}\frac{EF}{V_{P}}e^{-\frac{v_{P}}{v_{I}}\frac{EF}{V_{P}}t},$$

(39)


The spatially averaged interstitial concentration of the TK model, $\bar{C}_{I,TK}\left( t \right)$, is given by

$$\bar{C}_{I,TK}\left( t \right)=\frac{v_{P}}{v_{I}}\frac{F}{V_{P}}\left( \frac{1}{1-H_{LV}} \right)\left[ \gamma\left[ \left\{ N_{A1}\left( e^{-\frac{v_{P}}{v_{I}}\frac{EF}{V_{P}}t_{A1}}-e^{-\mu_{B,A}t_{A1}} \right)+N_{A2}t_{A1}e^{-\mu_{B,A}t_{A1}} \right\}u\left( t_{A1} \right)+\left( N_{A3}e^{-\frac{v_{P}}{v_{I}}\frac{EF}{V_{P}}t_{A2}}+N_{A4}e^{-\mu_{B,A}t_{A2}}+N_{A5}e^{-\mu_{G,A}t_{A2}}+N_{A6}t_{A2}e^{-\mu_{B,A}t_{A2}} \right)u\left( t_{A2} \right) \right]+\left( 1-\gamma\right)\left[ \left\{ N_{PV1}\left( e^{-\frac{v_{P}}{v_{I}}\frac{EF}{V_{P}}t_{PV1}}-e^{-\mu_{B,PV}t_{PV1}} \right)+N_{PV2}t_{PV1}e^{-\mu_{B,PV}t_{PV1}} \right\}u\left( t_{PV1} \right)+\left( N_{PV3}e^{-\frac{v_{P}}{v_{I}}\frac{EF}{V_{P}}t_{PV2}}+N_{PV4}e^{-\mu_{B,PV}t_{PV2}}+N_{PV5}e^{-\mu_{G,PV}t_{PV2}}+N_{PV6}t_{PV2}e^{-\mu_{B,PV}t_{PV2}} \right)u\left( t_{PV2} \right) \right] \right],$$

(40)


where $N_{A1}={Ea_{B,A}}/{\left( \mu_{B,A}-N_{1} \right)^{2}}$, $N_{A2}=-N_{A1}\left( \mu_{B,A}-N_{1} \right)$, $N_{A3}={N_{A1}a_{G,A}}/\left( \mu_{G,A}-N_{1} \right)$, $N_{A4}=N_{A3}\left\{ \left( \mu_{G,A}-N_{1} \right)/\left( \mu_{B,A}-\mu_{G,A} \right) \right\}\left\{ 1+\left( \mu_{B,A}-N_{1} \right)/\left( \mu_{B,A}-\mu_{G,A} \right) \right\}$, $N_{A5}=-N_{A3}\left\{ \left( \mu_{B,A}-N_{1} \right)/\left( \mu_{B,A}-\mu_{G,A} \right) \right\}^{2}$, $N_{A6}=-N_{A5}{\left( \mu_{G,A}-N_{1} \right)\left( \mu_{B,A}-\mu_{G,A} \right)}/\left( \mu_{B,A}-N_{1} \right)$, $N_{PV1}={Ea_{B,PV}}/{\left( \mu_{B,PV}-N_{1} \right)^{2}}$, $N_{PV2}=-N_{PV1}\left( \mu_{B,PV}-N_{1} \right)$, $N_{PV3}={N_{PV1}a_{G,PV}}/\left( \mu_{G,PV}-N_{1} \right)$, $N_{PV4}=N_{PV3}\left\{ \left( \mu_{G,PV}-N_{1} \right)/\left( \mu_{B,PV}-\mu_{G,PV} \right) \right\}\left\{ 1+\left( \mu_{B,PV}-N_{1} \right)/\left( \mu_{B,PV}-\mu_{G,PV} \right) \right\}$, $N_{PV5}=-N_{PV3}\left\{ \left( \mu_{B,PV}-N_{1} \right)/\left( \mu_{B,PV}-\mu_{G,PV} \right) \right\}^{2}$, $N_{PV6}=-N_{PV5}{\left( \mu_{G,PV}-N_{1} \right)\left( \mu_{B,PV}-\mu_{G,PV} \right)}/\left( \mu_{B,PV}-N_{1} \right)$, where $N_{1}=\left( {v_{P}}/{v_{I}} \right)\left( {EF}/{V_{P}} \right)$.

The TK model can be generalized to allow for an intravascular contribution in the tissue [21,22]. This generalization is known as the ETK model, in which the concentration in the tissue plasma is approximated by the net input function (i.e., AIF and PVIF) for the liver. The tissue residue function for the ETK model is

$$R_{T,ETK}\left( t \right)=\frac{V_{P}}{F}\delta\left( t \right)+R_{T,TK}\left( t \right).$$

(41)


The impulse response functions of the plasma and interstitial compartments of the ETK model, $Q_{P,ETK}\left( t \right)$ and $Q_{I,ETK}\left( t \right)$, are

$$Q_{P,ETK}\left( t \right)=\delta\left( t \right)$$

(42)


and

$$Q_{I,ETK}\left( t \right)=Q_{I,TK}\left( t \right),$$

(43)


The spatially averaged plasma and interstitial concentrations of the ETK model, $\bar{C}_{P,ETK}\left( t \right)$ and $\bar{C}_{I,ETK}\left( t \right)$ , are given by

$$\bar{C}_{P,ETK}\left( t \right)=\frac{\gamma C_{A}\left( t-t_{Lag,T} \right)+\left( 1-\gamma\right)C_{PV}\left( t-t_{Lag,T} \right)}{1-H_{LV}}$$

(44)


and

$$\bar{C}_{I,ETK}\left( t \right)=\bar{C}_{I,TK}\left( t \right).$$

(45)


In DCE-MRI, CA is detected indirectly via its effect of increasing the longitudinal relaxation rate in the surrounding water molecules. A tissue voxel is typically composed of four water-containing compartments, two cellular compartments (red blood cells (RBCs) and parenchyma cells) and two extracellular compartments (plasma and interstitial compartments). In general, it is accepted that water exchange between RBC and blood plasma is extremely rapid because of the high water permeability of the RBC membrane, and thus plasma and RBC can be considered as a single water compartment with a single T1. The decay of longitudinal magnetization $M_{q}$ in the whole blood, interstitial space, and parenchyma cells (with *q*=B, I, and C, respectively) can be described by the following coupled Bloch-McConnell equations [23]:

$$\frac{dM}{dt}=XM+C,$$

(46)


where $\mathbf{M}=\left[ M_{B}\left( t \right), M_{I}\left( t \right), M_{C}\left( t \right) \right]^{T}$ ($T$ denotes transpose), $\mathbf{C}=\left[ R_{10B}M_{0B}, R_{10I}M_{0I}, R_{10C}M_{0C} \right]^{T}$, and:

$$X=\left[ \begin{matrix} -\left( R_{10B}+K_{BI} \right) & K_{IB} & 0 \\ K_{BI} & -\left( R_{10I}+K_{IB}+K_{IC} \right) & K_{CI} \\ 0 & K_{IC} & -\left( R_{10C}+K_{CI} \right) \end{matrix} \right],$$

(47)


where $\mathbf{X}$ is the exchange matrix of the 3S2X model, and $K_{qr}$ denotes the rate of transfer of magnetization from compartment *q* to compartment *r*. $M_{0q}$ is the equilibrium magnetization in compartment *q*, and $R_{10q}$ ($=1/{T_{10q}}$) denotes the native longitudinal relaxation rate. Here it is assumed that the direct exchange of water between blood and parenchyma cells is negligible (i.e., $K_{\mathrm{BC}}\cong0$). Linear three-site exchange of water (i.e., 3S2X) between compartments *q* and *r* requires that [23]

$$K_{qr}M_{0q}=K_{rq}M_{0r}.$$

(48)


The $K_{qr}$ can also be expressed in terms of the water mean lifetimes ($\tau_{B}$, $\tau_{I}$, and $\tau_{C}$) and volume fractions of the three compartments by keeping the mass balance: $K_{\mathrm{BI}}=1/{\tau_{B}}$, $K_{\mathrm{IB}}=1/{\tau_{I}}-\left( {v_{C}}/{v_{I}} \right)/{\tau_{C}}$, $K_{\mathrm{IC}}=1/{\tau_{I}}-\left( {v_{B}}/{v_{I}} \right)/{\tau_{B}}$, and $K_{\mathrm{CI}}=1/{\tau_{C}}$, where $v_{B}={v_{P}}/\left( 1-H_{\mathrm{SV}} \right)$, and $H_{\mathrm{SV}}$ is the hematocrit in small vessels ($\cong$0.25) [10]. The three mean lifetimes are related by ${v_{I}}/{\tau_{I}}={v_{B}}/{\tau_{B}}+{v_{C}}/{\tau_{C}}$ [6]. Here we assume that the water fraction in each compartment is 1 for simplicity [2-4]. The longitudinal magnetization evolving from a 3S2X model can be expressed as a linear sum of the blood, interstitial, and cellular compartment longitudinal magnetizations (i.e., $M\left( t \right)=M_{B}\left( t \right)+M_{I}\left( t \right)+M_{C}\left( t \right)$). The cellular-interstitial water exchange rate is related to ${PS}_{C}$. The cell-to-interstitium water transfer rate, $K_{\mathrm{CI}}$, is given by [7]

$$K_{CI}=\frac{1}{\tau_{C}}=60\frac{{PS}_{C}}{V_{C}}=60\frac{{PS}_{C}}{v_{C}V_{T}},$$

(49)


where $V_{C}$ and ${PS}_{C}$ are defined in Table 1. The constant 60 is multiplied so that the time-scale is converted from minutes to seconds. On the other hand, the vascular-interstitial water exchange rate is related to $PS$ (or $EF$). According to the physiologic assumptions of each kinetic model, CA is exchanged between the plasma and interstitial compartments by $EF$ in the ETK and AATH models and by $PS$ in the 2CX and DP models. Thus, the blood-to-interstitium water transfer rate, $K_{\mathrm{BI}}$, is constrained with parameters to take into account the vascular-interstitial CA exchange for each kinetic model:

$$K_{BI}=\frac{1}{\tau_{B}}=\left\{ \begin{matrix} 60\left( 1-H_{SV} \right)\frac{PS}{V_{P}} & for WX-2CX and WX-DP models \\ 60\left( 1-H_{SV} \right)\frac{EF}{V_{P}} & for WX-ETK and WX-AATH models \end{matrix} \right.$$

(50)


Note that $K_{\mathrm{BI}}$ (or $K_{\mathrm{IB}}$) does not apply in the WX-TK model because it assumes that $v_{P}\ll v_{I}$ in the estimate of $C_{T}\left( t \right)$ (and thus neglects $\bar{C}_{P}\left( t \right)$) [1]. According to the formalism presented in Equations (49) and (50), the interstitium-to-cell water transfer $K_{\mathrm{IC}}$ and the interstitium-to-blood water transfer $K_{\mathrm{IB}}$ can be given by

$$K_{IC}=\frac{1}{\tau_{I}}-\frac{v_{B}}{v_{I}}\frac{1}{\tau_{B}}=60\frac{{PS}_{C}}{V_{I}}=60\frac{{PS}_{C}}{v_{I}V_{T}}$$

(51)


and

$$K_{IB}=\frac{1}{\tau_{I}}-\frac{v_{C}}{v_{I}}\frac{1}{\tau_{C}}=\left\{ \begin{matrix} 60\frac{PS}{V_{I}}=60\frac{v_{P}}{v_{I}}\frac{PS}{V_{P}} & for WX-2CX and WX-DP models \\ 60\frac{EF}{V_{I}}=60\frac{v_{P}}{v_{I}}\frac{EF}{V_{P}} & for WX-ETK and WX-AATH models \end{matrix} \right..$$

(52)


In the absence of CA, it is assumed that the water exchange system is in the FXL [1]. The FXL condition is also maintained for estimation of the postcontrast relaxation rate $R_{1}\left( t \right)$ only in the dual feeding vessels during CA passage because both consist of a single blood pool. Under the FXL condition, T1 after the administration of paramagnetic CA can be replaced by: $R_{1}\left( t \right)=1/{T_{1}\left( t \right)}=R_{10}+r_{1}C_{T}\left( t \right)=1/{T_{10}}+r_{1}C_{T}\left( t \right)$, where $C_{T}\left( t \right)$ is the tissue concentration, $r_{1}$ is the spin-lattice relaxivity, $R_{1}\left( t \right)$ (in sec^-1^) and $R_{10}$ (in sec^-1^) are the post- and precontrast relaxation rates, and $T_{1}\left( t \right)$ (in sec) and $T_{10}$ (in sec) are the post- and precontrast (native) T1 values, respectively. The relaxivities in tissue are assumed to be equal to those in aqueous solution ($r_{1}=$4.5 sec^-1^ mM^-1^, $r_{2}=$5.5 sec^-1^ mM^-1^ at 21°C and 1.5 T [24], where $r_{2}$ is the spin-spin relaxivity). The signal intensity obtained from a spoiled gradient echo sequence in the FXL as given by the Ernst-Anderson equation is [25]

$$S_{T}\left( t \right)=g\cdot PD\cdot e^{-TE\left\{ \frac{1}{T_{20}^{*}}+r_{2}C_{T}\left( t \right) \right\}}sin\left( \theta\right)\frac{1-e^{-TR\cdot R_{1}\left( t \right)}}{1-cos\left( \theta\right)e^{-TR\cdot R_{1}\left( t \right)}},$$

(53)


where $g$ is the machine gain, $PD$ is the proton density, $T_{20}^{*}$ is the precontrast effective T2 value, $\theta$ is the flip angle, and $S_{T}\left( t \right)$ is the MR signal intensity in the tissue at a time $t$. Note that MR signal intensities in the feeding vessels can also be expressed by replacing of $C_{T}\left( t \right)$ with $C_{A}\left( t \right)$ or $C_{\mathrm{PV}}\left( t \right)$ in Equation (53). The native T1 ($T_{10}$) value can be calculated by use of the preconstrast MR images with variable flip angles [26]. Rearranging Equation (53) with $t=C_{T}\left( t \right)=0$ yields

$$Y=e^{-\frac{TR}{T_{10}}}\cdot X-g\cdot PD\cdot\left( 1-e^{-\frac{TR}{T_{10}}} \right)e^{-\frac{TE}{T_{20}^{*}}},$$

(54)


where $Y={S_{T}\left( 0 \right)}/{\sin\left( \theta\right)}$, $X={S_{T}\left( 0 \right)}/{\tan\left( \theta\right)}$, and $S_{T}\left( 0 \right)$ is the precontrast signal intensity measured for a flip angle $\theta$. Hence, a plot of $Y$ against $X$ for a range of flip angles results in a straight line, and $T_{10}$ can be calculated from the slope. In the FXL, the entire tissue relaxes with a single effective $R_{1}\left( t \right)$ representing the weighted average of the three-compartmental $R_{1}$s, i.e., $R_{1}\left( t \right)=v_{B}R_{1B}\left( t \right)+v_{I}R_{1I}\left( t \right)+v_{C}R_{10C}$, where $R_{1B}\left( t \right)$ and $R_{1I}\left( t \right)$ denote the postcontrast longitudinal relaxation rate within the blood and that within the interstitial space, respectively. Likewise, in the absence of CA, $R_{10C}$ for the WX model is calculated by: $R_{10C}=\left( R_{10}-v_{I}R_{10I} \right)/{v_{C}}$ for the 2SX model [1], and

$R_{10C}=\left( R_{10}-v_{B}R_{10B}-v_{I}R_{10I} \right)/{v_{C}}$ for the 3S2X model [5], where $R_{10B}$ and $R_{10I}$ are assumed to be 0.74 and 0.5 s^-1^, respectively [3,5].

The effect of CA in the blood and interstitial spaces can be incorporated into the exchange matrix $\mathbf{X}$ by replacement of $R_{10B}$ and $R_{10I}$ in Equation (47) with $R_{1B}\left( t \right)=r_{1B}\left( 1-H_{\mathrm{SV}} \right)\bar{C}_{P}\left( t \right)+R_{10B}$ and $R_{1I}\left( t \right)=r_{1I}\bar{C}_{I}\left( t \right)+R_{10I}$, respectively, where $r_{1B}$ and $r_{1I}$ are the respective relaxivities [6], which are set to a constant, i.e., $r_{1B}=r_{1I}=r_{1}$. For a spoiled gradient echo acquisition, the Ernst-Anderson equation that uses $\mathbf{X}$ from Equation (47) is applied to finding the signal in each compartment [5,6]:

$$S=g\cdot PD\cdot e^{-TE\left\{ \frac{1}{T_{20}^{*}}+r_{2}C_{T}\left( t \right) \right\}}sin\left( \theta\right)\left[ I-cos\left( \theta\right)e^{TR\cdot X} \right]^{-1}\left( I-e^{TR\cdot X} \right)V,$$

(55)


where $\mathbf{S}=\left[ S_{B}\left( t \right), S_{I}\left( t \right), S_{C}\left( t \right) \right]^{T}$, $\mathbf{V}=\left[ v_{B}, v_{I}, v_{C} \right]^{T}$, $\mathbf{I}$ is the 3$\times$3 identity matrix, $e^{TR\cdot\mathbf{X}}$ is a matrix exponential, and the transverse relaxation rate, $R_{2}^{*}\left( t \right)=1/{T_{20}^{*}}+r_{2}C_{T}\left( t \right)$, is assumed to be under the FXL condition for simplicity because the T1 effects are of primary concern in this study. The matrix exponential can be calculated based on a scaling and squaring algorithm with a Pade approximation method [27]. To have no dependence on $T_{20}^{*}$, the relative signal enhancement in the tissue, $E_{T}\left( t \right)$, can be used as an objective function for curve-fitting of DCE-MRI data, i.e.,

$$E_{T}\left( t \right)=\frac{S_{B}\left( t \right)+S_{I}\left( t \right)+S_{C}\left( t \right)}{S_{B}\left( 0 \right)+S_{I}\left( 0 \right)+S_{C}\left( 0 \right)}-1.$$

(56)


Therefore, $E_{T}\left( t \right)$ is fitted by use of Equations (54) to (56), with $\bar{C}_{P}\left( t \right)$, $\bar{C}_{I}\left( t \right)$, and $C_{T}\left( t \right)$ for the 3S2X model (i.e., WX-ETK, WX-2CX, WX-AATH, and WX-DP models).

When $v_{P}\ll v_{I}$ is assumed, the 3S2X model can be simplified by ignoring any contribution from CA in the plasma, and thus the 2SX model can be used. This system can be described by use of a two-pool exchange formalism [2]. The solution has a bi-exponential form with the T1 relaxation of the system described by two rate constants, $R_{1S}$ and $R_{1L}$, where $R_{1S}$ is the rate constant for the component with the smaller T1 ($T_{1S}=1/{R_{1S}}$), and $R_{1L}$ is the rate constant for the component with the larger T1 ($T_{1L}=1/{R_{1L}}$), and their respective fractional apparent populations, $a_{L}$ and $a_{S}$, where $a_{S}+a_{L}=1$. For the 2SX model, a closed-form expression of MR signal intensity is given by [1,2]

$$S_{T}\left( t \right)=g\cdot PD\cdot e^{-TE\left\{ \frac{1}{T_{20}^{*}}+r_{2}C_{T}\left( t \right) \right\}}sin\left( \theta\right)\left\{ a_{S}\left( \frac{1-e^{-TR\cdot R_{1S}\left( t \right)}}{1-cos\left( \theta\right)e^{-TR\cdot R_{1S}\left( t \right)}} \right)+a_{L}\left( \frac{1-e^{-TR\cdot R_{1L}\left( t \right)}}{1-cos\left( \theta\right)e^{-TR\cdot R_{1L}\left( t \right)}} \right) \right\},$$

(57)


with

$$\left( \begin{matrix} R_{1S}\left( t \right) \\ R_{1L}\left( t \right) \end{matrix} \right)=\frac{1}{2}\left\{ 2R_{10C}+r_{1I}\bar{C}_{I}\left( t \right)+\frac{R_{10}-R_{10C}+K_{CI}}{v_{I}}\pm\sqrt{\left( 2K_{CI}-r_{1I}\bar{C}_{I}\left( t \right)-\frac{R_{10}-R_{10C}+K_{CI}}{v_{I}} \right)^{2}+4K_{CI}K_{IC}} \right\}$$

(58)


and

$$a_{S}\left( t \right)=\frac{1}{2}-\frac{1}{2}\left\{ \frac{\left( \frac{R_{10C}-R_{10}}{v_{I}}-r_{1I}\bar{C}_{I}\left( t \right) \right)\left( v_{I}-v_{C} \right)+\frac{K_{CI}}{v_{I}}}{\sqrt{\left( 2K_{CI}-r_{1I}\bar{C}_{I}\left( t \right)-\frac{R_{10}-R_{10C}+K_{CI}}{v_{I}} \right)^{2}+4K_{CI}K_{IC}}} \right\}.$$

(59)


The relative signal enhancement in the tissue, $E_{T}\left( t \right)$, is given by

$$E_{T}\left( t \right)=\frac{S_{T}\left( t \right)}{S_{T}\left( 0 \right)}-1.$$

(60)


Therefore, $E_{T}\left( t \right)$ is fitted by use of Equation (54), and by (57) to (60), with $\bar{C}_{I}\left( t \right)$ and $C_{T}\left( t \right)$ for the 2SX model (i.e., the WX-TK model).

The parameters that can be estimated directly by parametric fitting with the five different WX tracer kinetic models are $F/{V_{P}}$, $\gamma$, ${PS}/{V_{P}}$, $v_{P}$, $v_{I}$, $\tau_{C}$, and $t_{Lag,T}$ (note that $v_{B}+v_{I}+v_{C}=1$, $F/{V_{T}}={v_{P}F}/{V_{P}}$, and ${PS}/{V_{T}}={v_{P}PS}/{V_{P}}={v_{I}PS}/{V_{I}}$) [28]. Therefore, the $BF$, ${BF}_{A}$, ${BF}_{\mathrm{PV}}$, $BV$, $MTT$, and $PS$ can be computed according to: $BV={{100\cdot V}_{P}}/\left\{ \left( 1-H_{\mathrm{SV}} \right)\cdot m \right\}={{100\cdot v}_{P}}/\left\{ \left( 1-H_{\mathrm{SV}} \right)\cdot\rho_{T} \right\}$ (in mL/100 g), and $m=\rho_{T}V_{T}$ is the mass of the tissue with density $\rho_{T}$ ($=$1.04 g/cm^3^ in the case of soft tissues), $BF=BV\cdot F/{V_{P}}$ (in mL/min/100 g), ${BF}_{A}=\gamma BF$ (in mL/min/100 g), ${BF}_{\mathrm{PV}}=\left( 1-\gamma\right)BF$ (in mL/min/100 g), $MTT=\left( V_{P}+V_{I} \right)/F$ (in min) ($MTT={V_{I}}/F$ for the WX-TK model), and $PS=\left( 1-H_{\mathrm{SV}} \right)\cdot BV\cdot{PS}/{V_{P}}$ (in mL/min/100 g).

**References**

1. Yankeelov TE, Rooney WD, Li X, Springer CS,Jr. Variation of the relaxographic "shutter-speed" for transcytolemmal water exchange affects the CR bolus-tracking curve shape. Magn Reson Med. 2003;50: 1151-1169. doi: 10.1002/mrm.10624.

2. Buckley DL, Kershaw LE, Stanisz GJ. Cellular-interstitial water exchange and its effect on the determination of contrast agent concentration in vivo: dynamic contrast-enhanced MRI of human internal obturator muscle. Magn Reson Med. 2008;60: 1011-1019. doi: 10.1002/mrm.21748; 10.1002/mrm.21748.

3. Paudyal R, Poptani H, Cai K, Zhou R, Glickson JD. Impact of transvascular and cellular-interstitial water exchange on dynamic contrast-enhanced magnetic resonance imaging estimates of blood to tissue transfer constant and blood plasma volume. J Magn Reson Imaging. 2013;37: 435-444. doi: 10.1002/jmri.23837; 10.1002/jmri.23837.

4. Zhang J, Kim S. Uncertainty in MR tracer kinetic parameters and water exchange rates estimated from T -weighted dynamic contrast enhanced MRI. Magn Reson Med. 2013. doi: 10.1002/mrm.24927; 10.1002/mrm.24927.

5. Bains LJ, McGrath DM, Naish JH, Cheung S, Watson Y, Taylor MB, et al. Tracer kinetic analysis of dynamic contrast-enhanced MRI and CT bladder cancer data: A preliminary comparison to assess the magnitude of water exchange effects. Magn Reson Med. 2010;64: 595-603. doi: 10.1002/mrm.22430; 10.1002/mrm.22430.

6. Li X, Rooney WD, Springer CS,Jr. A unified magnetic resonance imaging pharmacokinetic theory: intravascular and extracellular contrast reagents. Magn Reson Med. 2005;54: 1351-1359. doi: 10.1002/mrm.20684.

7. Landis CS, Li X, Telang FW, Coderre JA, Micca PL, Rooney WD, et al. Determination of the MRI contrast agent concentration time course in vivo following bolus injection: effect of equilibrium transcytolemmal water exchange. Magn Reson Med. 2000;44: 563-574.

8. Koh TS, Bisdas S, Koh DM, Thng CH. Fundamentals of tracer kinetics for dynamic contrast-enhanced MRI. J Magn Reson Imaging. 2011;34: 1262-1276. doi: 10.1002/jmri.22795; 10.1002/jmri.22795.

9. Orton MR, d'Arcy JA, Walker-Samuel S, Hawkes DJ, Atkinson D, Collins DJ, et al. Computationally efficient vascular input function models for quantitative kinetic modelling using DCE-MRI. Phys Med Biol. 2008;53: 1225-1239. doi: 10.1088/0031-9155/53/5/005; 10.1088/0031-9155/53/5/005.

10. Brix G, Griebel J, Kiessling F, Wenz F. Tracer kinetic modelling of tumour angiogenesis based on dynamic contrast-enhanced CT and MRI measurements. Eur J Nucl Med Mol Imaging. 2010;37 Suppl 1: S30-51. doi: 10.1007/s00259-010-1448-7.

11. Koh TS, Thng CH, Lee PS, Hartono S, Rumpel H, Goh BC, et al. Hepatic metastases: in vivo assessment of perfusion parameters at dynamic contrast-enhanced MR imaging with dual-input two-compartment tracer kinetics model. Radiology. 2008;249: 307-320. doi: 10.1148/radiol.2483071958.

12. Sourbron SP, Buckley DL. Tracer kinetic modelling in MRI: estimating perfusion and capillary permeability. Phys Med Biol. 2012;57: R1-33. doi: 10.1088/0031-9155/57/2/R1; 10.1088/0031-9155/57/2/R1.

13. Johnson JA, Wilson TA. A model for capillary exchange. Am J Physiol. 1966;210: 1299-1303.

14. St Lawrence KS, Lee TY. An adiabatic approximation to the tissue homogeneity model for water exchange in the brain: I. Theoretical derivation. J Cereb Blood Flow Metab. 1998;18: 1365-1377. doi: 10.1097/00004647-199812000-00011.

15. Koh TS, Cheong LH, Hou Z, Soh YC. A physiologic model of capillary-tissue exchange for dynamic contrast-enhanced imaging of tumor microcirculation. IEEE Trans Biomed Eng. 2003;50: 159-167. doi: 10.1109/TBME.2002.807657.

16. Koh TS. On the a priori identifiability of the two-compartment distributed parameter model from residual tracer data acquired by dynamic contrast-enhanced imaging. IEEE Trans Biomed Eng. 2008;55: 340-344. doi: 10.1109/TBME.2007.910682; 10.1109/TBME.2007.910682.

17. Sourbron SP, Buckley DL. On the scope and interpretation of the Tofts models for DCE-MRI. Magn Reson Med. 2011;66: 735-745. doi: 10.1002/mrm.22861; 10.1002/mrm.22861.

18. Thompson MD, Beard DA. Physiologically based pharmacokinetic tissue compartment model selection in drug development and risk assessment. J Pharm Sci. 2012;101: 424-435. doi: 10.1002/jps.22768; 10.1002/jps.22768.

19. Renkin EM. Transport of potassium-42 from blood to tissue in isolated mammalian skeletal muscles. Am J Physiol. 1959;197: 1205-1210.

20. Crone C. The permeability of capillaries in various organs as determined by use of the 'indicator diffusion' method. Acta Physiol Scand. 1963;58: 292-305. doi: 10.1111/j.1748-1716.1963.tb02652.x.

21. Tofts PS, Brix G, Buckley DL, Evelhoch JL, Henderson E, Knopp MV, et al. Estimating kinetic parameters from dynamic contrast-enhanced T(1)-weighted MRI of a diffusable tracer: standardized quantities and symbols. J Magn Reson Imaging. 1999;10: 223-232.

22. Tofts PS. Modeling tracer kinetics in dynamic Gd-DTPA MR imaging. J Magn Reson Imaging. 1997;7: 91-101.

23. Spencer RG, Fishbein KW. Measurement of spin-lattice relaxation times and concentrations in systems with chemical exchange using the one-pulse sequence: breakdown of the Ernst model for partial saturation in nuclear magnetic resonance spectroscopy. J Magn Reson. 2000;142: 120-135. doi: 10.1006/jmre.1999.1925.

24. Tofts PS, Berkowitz B, Schnall MD. Quantitative analysis of dynamic Gd-DTPA enhancement in breast tumors using a permeability model. Magn Reson Med. 1995;33: 564-568.

25. Wehrli FW. Fast-Scan Magnetic Resonance: Principles and Applications. 1st ed. New York: Raven Press; 1991.

26. Fram EK, Herfkens RJ, Johnson GA, Glover GH, Karis JP, Shimakawa A, et al. Rapid calculation of T1 using variable flip angle gradient refocused imaging. Magn Reson Imaging. 1987;5: 201-208.

27. Higham NJ. The scaling and squaring method for the matrix exponential revisited. Siam J Matrix Anal Appl. 2005;26: 1179-1193.

28. Brix G, Bahner ML, Hoffmann U, Horvath A, Schreiber W. Regional blood flow, capillary permeability, and compartmental volumes: measurement with dynamic CT--initial experience. Radiology. 1999;210: 269-276.
